# Clusters of 24-hour movement behavior and diet and their relationship with health indicators among youth: a systematic review

**DOI:** 10.1186/s12889-024-18364-6

**Published:** 2024-04-18

**Authors:** Gabrielli T. de Mello, Giseli Minatto, Rafael M. Costa, Rebecca M. Leech, Yingting Cao, Rebecca E. Lee, Kelly S. Silva

**Affiliations:** 1 Research Center for Physical Activity and Health, Federal University of Santa Catarina, Florianópolis, Brazil; 2 Institute for Physical Activity and Nutrition (IPAN), Deakin University, Melbourne, Australia; 3 School of Allied Health, Human Services and Sport, La Trobe University, Melbourne, Australia; 4 Center for Health Promotion and Disease Prevention, Edson College of Nursing and Health Innovation, Arizona State University, Phoenix, USA

**Keywords:** Adolescent health, Child Health, Clustering, Diet, Food and Nutrition, Exercise, Sleep

## Abstract

Movement-related behaviors (physical activity [PA], sedentary behavior [SB], and sleep) and diet interact with each other and play important roles in health indicators in youth. This systematic review aimed to investigate how PA, SB, sleep, and diet cluster in youth by biological sex; and to examine which cluster are associated with health indicators. This study was registered in PROSPERO (number: CRD42018094826). Five electronic databases were assessed. Eligibility criteria allowed studies that included youth (aged 19 years and younger), and only the four behaviors {PA, SB, sleep, and diet (ultra-processed foods [UPF]; fruits and vegetables [FV])} analyzed by applying data-based cluster procedures. From 12,719 articles screened; 23 were included. Of these, four investigated children, and ten identified clusters by biological sex. Sixty-six mixed cluster were identified including, 34 in mixed-sex samples, 10 in boys and 11 in girls. The most frequent clusters in mixed-sex samples were “High SB UPF Low Sleep”, “Low PA High SB Satisfactory Sleep”, and “High PA”. The main difference in profiles according to sex was that girls’ clusters were characterized by high sleep duration, whereas boys’ clusters by high PA. There were a few associations found between cluster types and health indicators, highlighting that youth assigned to cluster types with low PA exhibited higher adiposity. In conclusion, the youth presented a range of clusters of behaviors, typically exhibiting at least one unhealthy behavior. Similar patterns were observed in both sexes with the biggest difference in time of sleep for girls and PA for boys. These findings underscore the importance of intervention strategies targeting multiple behaviors simultaneously to enhance health risk profiles and indicators in children and adolescents.

**Supplementary Information:**

The online version contains supplementary material available at 10.1186/s12889-024-18364-6.

## Introduction

The 24-hour movement behaviors (i.e., physical activity—PA, sedentary behavior—SB, and sleep) and diet are referred to as energy balance-related behaviors (EBRBs) and mutually moderate each other's health impacts [1, 2]. For instance, the positive effects of engaging in regular PA or consuming fruit and vegetables might be compromised if individuals engage in prolonged periods of SB, exhibit short sleep duration, or consume ultra-processed foods. These EBRBs interact with each other and operate within a complex feedback loop, influencing body homeostasis, hormonal regulation, cellular metabolism, and impacting overall health in children and adolescents [1, 3, 4]. Youth engage in multiple risk behaviors simultaneously [5–7], which increases the risk of chronic diseases and all-cause mortality over and above the addictive effects of individual behaviors [8]. Thus, understanding how these interactions occur or how these behaviors cluster in the pediatric population may be promising for guiding future behavior change strategies that support healthy development [9]. Initiatives to support healthy behaviors for children and adolescents are crucial once risk behaviors emerge during youth and could interrupt the trajectory toward poor adult health [10].

Systematic reviews investigating the clustering of the 24-hour movement behaviors and diet have been previously conducted among youth [5, 7, 11–19]. However, it is noteworthy that all these behaviors were not investigated simultaneously among youth, except the review conducted by Liberali et al. [16]. Nevertheless, this study also included additional behaviors, such as tobacco and alcohol consumption, in its eligibility criteria. This could introduce variability in the types of clusters observed and their relationships with physical and mental health outcomes. Additionally, recent reviews [16–18] have advocated for future research to examine the clustering of PA, SB, sleep, and diet, given their robust correlations and interdependencies in shaping the lifestyle of children and adolescents.

It is known that the most frequently identified behavior profiles among youth were “high SB and consumption of ultra-processed foods”, “high PA”, “low PA and SB”, “low PA high/low SB”, and “low PA high SB” [5, 7, 14]. Studies also demonstrated that the intrapersonal characteristic, biological sex, may influence the adoption of behaviors [5, 20, 21]. For example, there are differences between boys and girls in the adoption of specific behaviors that may be explained by biological factors, such as hormonal and maturation differences, psychosocial factors, also by expectations, and social norms regarding behavior [22, 23]. Indeed, boys have presented in clusters characterized by high PA [7, 14, 24, 25] and tend to be in clusters with high amounts of screen time (e.g., watching television, using computers, and playing video games), whereas girls spend more time in social activities such as sitting and talking with their friends [7, 26–33] as well as had better diet quality (e.g., higher fruit and vegetables and lower ultra-processed foods consumption) when compared to boys [34, 35]. No difference has been observed in sleep time and clusters types between boys and girls [36–38].

In addition, clustering of behaviors have been associated with different health indicators [7, 14]. For example, children exhibiting unhealthy patterns—defined by at least two behaviors among poor diet quality, PA, high SB, and inadequate sleep—were more likely to have higher adiposity compared to their peers following healthy or mixed patterns [14]. In addition, children and adolescents in high PA and high SB clusters had higher body mass index and obesity [7]. Also, adolescents in clusters with more screen time, shorter sleep duration, and higher consumption of ultra-processed foods had higher insulin resistance, and girls in clusters with high screen time and an unhealthy lifestyle were at increased risk for being overweight [39]. In contrast, youths in clusters with more time spent in moderate and vigorous PA had lower insulin resistance, total high-density lipoprotein, and low-density lipoprotein cholesterol [40, 41].

Exploring how 24-h movement behaviors and diet cluster together and their association with diverse health indicators holds the potential to offer insights into the etiology of mental, physical, and overall health and wellbeing in children and adolescents [42]. This study advances previous investigations [5, 12–14] by conducting a systematic review designed to explore the clustering profiles of PA, SB, sleep, and diet in children and adolescents. Additionally, this study aims to explore these clusters according to biological sex and to examine which cluster types are associated with a variety of mental and physical health indicators. Understanding how these elements interact provides a holistic view of the determinants of health in young people, highlighting the need for effective interventions that simultaneously address these behaviors.

## Methods

This systematic review was registered in PROSPERO (registration number: CRD42018094826) and forms part of a large project on a global panorama of research examining the clustering of behaviors in children and adolescents. This article synthesized the evidence on clusters of PA, SB, sleep, and diet in youth and was reported considering the Preferred Reporting Items for Systematic Reviews and Meta-Analysis (PRISMA) guidelines [43, 44] and the extension Synthesis Without Meta-analysis (SWiM) [45] (see Supplementary material Table S1 and S2). The research question was formulated using the PI(E)COS framework, encompassing the Population (children and adolescents aged 19 years or younger), Intervention/Exposure (PA, SB, sleep and diet cluster types), Comparison/Control (not applicable), Outcome (physical and mental health outcomes), and Study design (any). The PI(E)COS question focused on identifying the types of behavioral clusters related to PA, SB, sleep, and diet, as well as exploring their relationships with physical and mental health outcomes in children and adolescents.

### Eligibility criteria

The criteria used to determine eligibility of each article for inclusion were: 1. included children and/or adolescents aged 19 years and younger or mean age between this range; 2. had undertaken a person-oriented statistical approach to identify clustering behaviors; 3. included only the four behaviors (PA, SB, sleep, and diet) in the analyzes; and 4. were written in English, Portuguese, or Spanish language. Original articles provided by any designs were included. Studies exclusively targeting clinical populations (e.g., disabilities, metabolic and/or cardiovascular diseases, population reached in hospitals) or derived clusters that included other behaviors or variables (e.g., tobacco use, unprotected sex, body mass index) were excluded.

### Search strategies and screening

This study involved a wide behavior search strategy with no restrictions of publication year, including studies published in English, Portuguese and Spanish. Papers published up to and including May 2018 were identified through five electronic databases: PubMed, Scopus, Web of Science, LILACS, and PsycINFO. This search was then updated at the begin of April 2023. The search was independently conducted by two authors (GTM/RMC). When the articles matched, the metadata were exported and inserted into the Rayyan tool, where duplicates were removed before the screening process. The search encompassed sets of descriptors associated with behaviors (e.g., diet), person-oriented statistical approaches (e.g., 'cluster analysis'), and specific populations (e.g., adolescents*) (see Supplementary material Table S3). Terms within each search string were separated by the OR operator considering particularities from each database and Boolean operators and truncation symbols ($, * or ""). The search terms were based on pre-existing systematic reviews and then expanded with a Medical Subject Headings (MeSH) Browser search (https://meshb.nlm.nih.gov/). Reference lists of included studies and previous reviews were examined to identify any additional relevant articles.

The searches and screening were conducted to guarantee the inclusion of papers that investigated clustering behaviors of at least two of the behaviors among PA, SB, and/or diet. However, during the thorough reading stage, a selection of the studies that included only sleep, in addition to PA, SB, diet, met the inclusion criteria. Titles and abstracts were screened using the Rayyan tool, followed by independent full-text assessments using PDF reader software by two authors (GTM/RMC). Discrepancies were resolved by GM.

### Quality assessment

Risk of bias and methodological quality of included studies was assessed using a 17-point adapted version of the Quality Assessment Tool for Quantitative Studies of Effective Public Health Practice Project (EPHPP) [46]. This tool considers the following four methodological domains:

- 1) Selection bias, measured by the question “Are the individuals selected to participate in the study likely to be representative of the target population?”. Response possibilities were ≥ 80% = *strong* or 1; 79—60% = *moderate* or 0; ≤ 60% = *weak* or -1;
- 2) Study design, measured by the questions “Is there a description of the representativeness of the sample?”, with answer options: yes = 1; no = 0; “Was the sampling method described?”, with answer options: yes = 1; no = 0; “Was the method appropriate?”, with answer options: random = 1; not described = 0; convenience = -1. The *strong* classification was assigned for 1 in all three items, *moderate* for combinations: 1–1-0, 1–0-1, 1–0-0, and 0–0-1, and *weak* for all other combinations.
- 3) Information about instruments to evaluate PA, SB, sleep, and diet (Is there a prior validation report of the tool? yes = 1; no = 0); and information that would enable reproducing PA, SB, sleep, and diet assessment (Is there information that makes it possible to replicate the tool? yes = 1; no = 0). Studies using accelerometer to measure PA and/or SB and/or sleep were assigned a score of "1", that is, it was considered that there was a previous validation report of the instrument (*strong* for 1 in both outcome items, and *weak* for all other combinations); and.
- 4) Flow of people throughout the study (Were withdrawals and drop-outs reported in terms of numbers and/or reasons per group? yes = 1; no = 0) and percentage of participants completing the study (Indicate the percentage of participants completing the study? ≥ 80% = 1 or strong; 60–79% = 0 or moderate; ≤ 59% = -1 or weak). The classification *strong* was applied for 1 in both items or 0 and 1, *moderate* for combinations 1 and 0 or 0 and 0, and *weak* for all other combinations.

The risk of bias classification (low [strong], moderate [moderate] and high [weak]) for each domain was performed based on study distribution (see Supplementary material Table S4). Two independent reviewers (GTM and GM) assessed the quality of all studies, and a third reviewer (RMC) was consulted for the discrepancies.

### Data extraction and synthesis

Data were extracted and input into a tailored spreadsheet by the same peers of the aforementioned full-text process. Data extracted from each study included publication year, study name, sample country; design; sample size and age, data driven analytic method, and number of clusters identified.

Information about the instruments used to measure PA, SB, sleep, and diet behaviors were extracted and classified as (1) Defined (validated instruments), (2) Undefined (reported question and/or response option and instrument reference), (3) Undefined-Reproducible (reported question and response options and not mention the reference), (4) Objective measurement (e.g., accelerometer), (see Supplementary material Table S5 and Table S6). Additionally, information regarding the variables' domain and components utilized in cluster analysis for each paper (e.g., leisure/habitual PA, time spent watching TV/computer use) were extracted and presented as Supplementary material (Table S6).

Cluster types of characteristics were extracted as mixed-sex when clusters were identified from total samples, considering boys and girls together. When studies identified clusters separately for boys and girls, the characteristics were categorized according to biological sex. The study authors' descriptions of the cluster types were utilized for extracting data on the cluster types. For example, if authors characterized a cluster with low time watching TV, low PA levels, and high sleep time, the cluster type was classified as “Low PA SB and High sleep”. When authors did not describe the cluster in the article text, quantitative data present in figures and tables were used to identify the cluster types. Also, the association between cluster types and physical and mental health indicators were extracted as positive ( +), negative (-), and no association (0).

Sleep was classified as High (> 13 h – 3–4 years old; > 11 h – 5–13 years old; > 10 h – 14–17 years old); Sufficient (10–13 h – 3–4 years old; 9–11 h – 5–13 years old; 8–10 h – 14–17 years old); and Low (< 10 h – 3–4 years old; < 9 h—5–13 years old; < 8 h – 14–17 years old) [47, 48]. Diet characteristics were named as ultra-processed foods (UPF) (i.e., snacks, sweetened beverages, excessive salty foods, candies, and fried meals), and fruits and vegetables (FV) (i.e., fruits, vegetables, fiber consumption, green salads). Variables that did not fit in UPF and FV parameters were defined as “Specific Diet” (e.g., milk and meat consumption). For instance, a cluster described as low consumption of snacks and soft drinks, high consumption of fruits and vegetables, and high time spent in PA and SB was labeled as “High PA SB FV and Low UPF*”*.

## Results

Studies using the same sample data were considered a single study, using the most recent publication. The authors of a particular study [49] did not respond to an email requesting clarification on the cluster results of their study. Consequently, this study was not included in the analysis of cluster characteristics and associations.

## Studies description

**Fig. 1:**
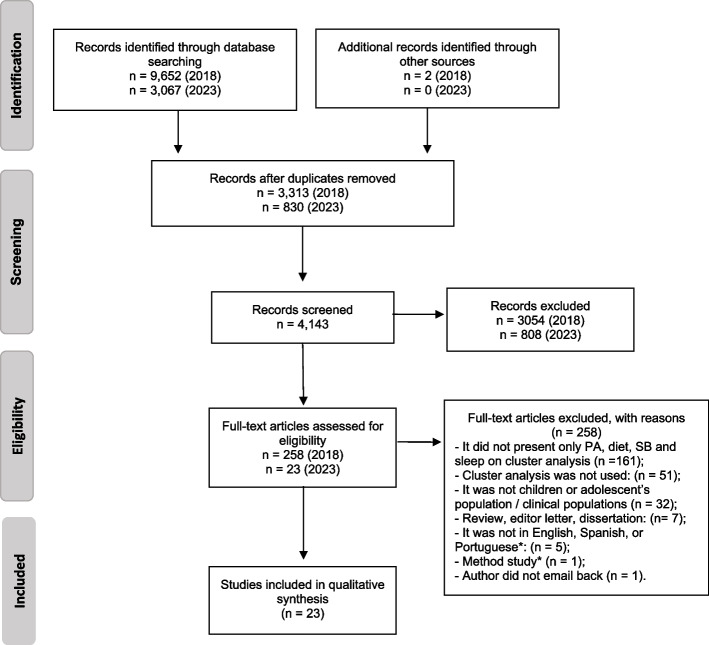
Flowchart of study inclusion for the review. PA: physical activity; SB: sedentary behavior. Note: * Polash idiom; Explained how to use cluster analysis – did not present results

A total of 12,719 articles were identified, of which 23 were included in this review following the exclusion of duplicates and through the screening process (Fig. 1). The majority of the studies were conducted in high-income European countries (70%) [20, 38–40, 50–56]. Two articles included data from an upper-middle income country (Brazil) [20, 57], and all other studies were from high-income countries. Of the 23 studies, 14 had a cross-sectional design [20, 36, 37, 39–41, 50, 52–55, 58–60], five carried out cross-sectional analyses of longitudinal [61–63] or experimental [38, 57] studies, and four had a longitudinal design [49, 51, 56, 64]. The study sample sizes varied from 235 [50] to 5,759 [36] participants, and most studies investigated adolescents, whereas four only investigated children [38, 51, 61, 63]. The analysis mostly used to identify clusters was k-means (*n* = 9), followed by principal components analysis (PCA) (*n* = 7), latent class analysis (LCA) (*n* = 2), and two-step cluster analysis (*n* = 2). Ten studies identified clusters according to biological sex. The number of clusters identified ranged from two [39, 54] to ten [20] in studies that analyzed mixed-sex samples; from two [50] to five [58] in boys, and from one [50] to five [58, 59] in girls (Table 1).

**Table 1:** Characteristics of studies included in the systematic review (*n* = 23)

Author (publication year)	Study name	Sample Country	Study Design	Sample size (% of girls)	Sample age (years; range or mean)	Data driven analytic method	Nº of clusters^DI^
Androutsos (2014) [40]	Healthy Growth	Greece	CS	2656 (50.1)	9–13	PCA	5
Collese (2018) [20]^**a**^	HELENA BRACAH	European^h^ Brazil	CS CS	1252 (52.7) 682 (54.2)	12.5–17.5 14.0–17.5	K-means^i^	10
Descarpentrie (2021) [64]^b^	EDEN	France	Longitudinal	978 (46.9)	5	PCA	Boys 3 / Girls 3
Descarpentrie (2022) [50]	ENFAMS	France	CS	235 (48.5)	6–12	PCA	Boys 2 / Girls 1
Descarpentrie (2023) [51]^b^	EDEN	France	Longitudinal	876 (47.6)	5	PCA	Boys 3 / Girls 3
D’ Souza (2021) [62]^c^	HAPPY	Australia	Longitudinal (CS analysis)	432 (43.5)	5.4–9.1 (7.6 ± 0.7)	K-means^i^, LPA, PCA	K-means^f^ 3, LPA 3, PCA 4
D’ Souza (2022) [61]^c^	HAPPY	Australia	Longitudinal (CS analysis)	432 (43.5)	5.4–9.1 (7.6 ± 0.7)	K-means^i^, LPA, PCA	K-means^f^ 3, LPA 3, PCA 4
Dumuid (2017) [37]	ISCOLE	Australia	CS	284 (53.9)	9–11	K-means^i^	4
Dumuid (2017) [36]	ISCOLE	Intercontinental^e^	CS	5759 (55.0)	9–11	K-means^i^	Boys 4 / Girls 4
Dumuid (2016) [58]	ISCOLE	Intercontinental^e^	CS	5710 (NR)	9–11	K-means^i^	Boys 5 / Girls 5
Fernández-Alvira (2013) [52]	ENERGY	European^f^	CS	5284 (54.0)	10–12	K-means^i^	Boys 4 / Girls 4
Ferrar and Golley (2015) [59]	NCNPAS	Australia	CS	1853 (49.8)	9–16	Two-steps	Boys 4 /Girls 5
Knebel (2022) [57]	*Movimente*	Brazil	CRCT (CS analysis)	750 (52.8%)	10–16 (13.1 ± 1.0)	Two-steps	3
Magee (2013) [63]	LSAC	Australia	Longitudinal (CS analysis)	1833 (48.4)	6–9	LCA	6
Miguel-Berges (2017) [38]	ToyBox	European^g^	RCT (CS analysis)	5387 (49.0)	3.5–5.5	K-means^i^	Boys 3 / Girls 4
Moraes (2016) [60]^**a**^	HELENA BRACAH	European^h^ Brazil	CS CS	1252 (52.7) 682 (54.2)	12.5–17.5 14 to 17.5	K-means^i^	5
Moschonis (2012) [41]^**d**^	Healthy Growth	Greece	CS	2043 (50.2)	9–13	PCA	5
Moschonis (2013) [53]^**d**^	Healthy Growth	Greece	CS	2073 (50.2)	9–13	PCA	Boys 3 / Girls 3
Nuutinen (2017) [39]	HBSC	Finland	CS	13 years: 2152 15 years: 2110	13 and 15	K-means	2
Pereira (2015) [54]	ISCOLE	Portugal	CS	686 (55.5)	9.5–10.5	LCA	2
Pérez-Rodrigo (2015) [55]	ANIBES Study	Spain	CS	415 (37.8)	9–17	K-means^i^	Boys 3 / Girls 4
Saldanha-Gomes (2020) [49]	EDEN	France	Longitudinal	2 years: 1436 (47.8%) / 5 years: 1195 (47%)	2 and 5	LCA	2 years (Boys 2 / Girls 2). 5 years (Boys 2 / Girls 4)
Wiersma (2022) [56]	GECKO	Netherlands	Longitudinal	1792	3–11	PCA	3

*NR* not reported, *LSAC* Longitudinal Study of Australian Children, *BRACAH* Brazilian Cardiovascular Adolescent Health, *EDEN* EDEN mother–child cohort study, *ENERGY* European Energy balance Research to prevent excessive weight Gain among Youth, *ENFAMS* *Enfants et familles sans logement*; *HAPPY* Healthy Active Preschool and Primary Years study, *HBSC* Health Behavior in School-aged Children, *HELENA* Healthy Lifestyle in Europe by Nutrition in Adolescence, *Movimente* Promotion of healthy lifestyles in adolescents and their relation to school performance—*Movimente* Study, *NCNPAS*, National Children’s Nutrition and Physical Activity Survey, *YRBSS* Youth Risk Behavior Surveillance System, *GECKO* Groningen Expert Center for Kids with Obesity, *LCA* Latent class analysis, *PCA* Principal component analysis, *RCT* randomized controlled trial, *CRCT* Cluster-randomized controlled trial, *CS* Cross-sectional

^DI^Detailed information about the cluster types is available in Supplementary Information Table S7

^a, b, c, d^clusters identified in same sample

^e^Australia, Brazil, Canada, China, Colombia, England, Finland, India, Kenya, Portugal, South Africa, and USA

^f^Belgium, Greece, Hungary, Netherlands, Norway, Slovenia, and Spain

^g^Belgium, Bulgaria, Germany, Greece, Poland, and Spain

^h^Austria, Belgium, France, Greece, Germany, Hungary, Italy, Spain, and Sweden

^i^With Wald’s method

**Fig. 2:**
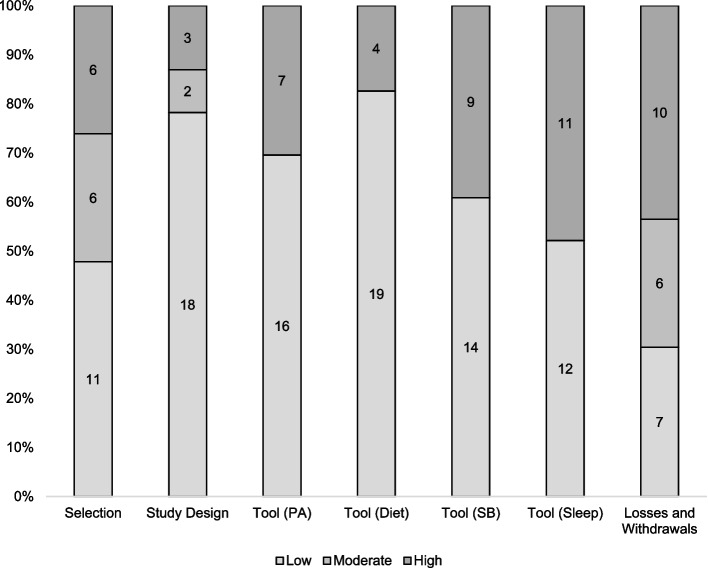
Assessment of the risk of bias of studies. PA: physical activity. SB: sedentary behavior. Low: low risk of bias. Moderate: moderate risk of bias. High: high risk of bias

Risk of bias assessment is presented in Fig. 2 and in Supplementary material Table S4. The percentage of disagreement among the risk of bias evaluators was 18.9% (kappa = -0.15; 1.0), ranging from 4.3% to 43.5%. Only one study [39] was classified with a low risk of bias for all evaluated criteria and three studies [38, 57, 58] showed moderate and low risk. The other studies showed a high risk of bias in at least one evaluated criterion and one study [63] presented a high risk of bias for all evaluated criteria (see Supplementary material Table S4). Six [20, 37, 60–63] of the 23 included studies failed to achieve at least 60% of the eligible response (response rate – selection bias) and 10 [20, 36, 37, 49, 51, 54, 55, 61–64] had ≤ 59% of participants who completed the study (participation rate – losses and withdrawals). The risk of bias for the study design was mostly low [39, 49–51, 54–59, 64] and moderate [36, 38, 40, 41, 52, 53]. Almost all studies presented information that would allow researchers to replicate the applied tool; however, a high risk of bias was found for the assessment of SB [36, 37, 40, 49, 51, 52, 56, 63, 64] and sleep [40, 41, 49–51, 53, 55, 56, 59, 63, 64].

### Data synthesis

#### Instruments and clusters outcomes

The most frequent instruments used to measure PA, SB, sleep, and diet were questionnaires with validation process (see Supplementary material Table S5). Diet also included three studies evaluated by a face-to-face 24 h dietary recall collected over two consecutive week days and one weekend day [40, 41, 53, 55]. Ten studies evaluated youth diet using food frequency questionnaires [20, 36–38, 50, 51, 54, 56, 58, 61].

A wide range of variables were included in data-driven cluster procedures (see Supplementary material Table S6). Most frequent PA outcomes involved minutes per day in moderate and vigorous PA [53, 54, 61, 62], and moderate [36, 37, 55, 58], and vigorous [36, 37, 55, 58] PA only. Most studies included screen time defined by hours per day spent on television, computer and videogame [20, 36, 37, 40, 41, 50, 51, 53, 54, 64]. Sleep outcomes mainly involved self-reported hours of sleep duration per day [20, 38–40, 50–53, 55, 57, 63], and only one study assessed sleep quality and discrepancy (time sleeping on weekend vs. week nights) [39], and regular bedtime and wake-up time [49]. The most frequent outcomes of diet used were intakes of fruit and vegetables measured separately [40, 41, 50, 51, 53, 59, 61, 62, 64] and sweet/sugary beverages [20, 38, 40, 41, 50–54, 60, 63, 64]. Five studies also used PCA [36, 37, 57, 58] and factor analysis [55] to determine dietary patterns based on 5 to 38 different food groups. Outcomes of eating habits included breakfast and meal frequency [40, 41, 53] were also identified.

#### Clusters Types

**Fig. 3:**
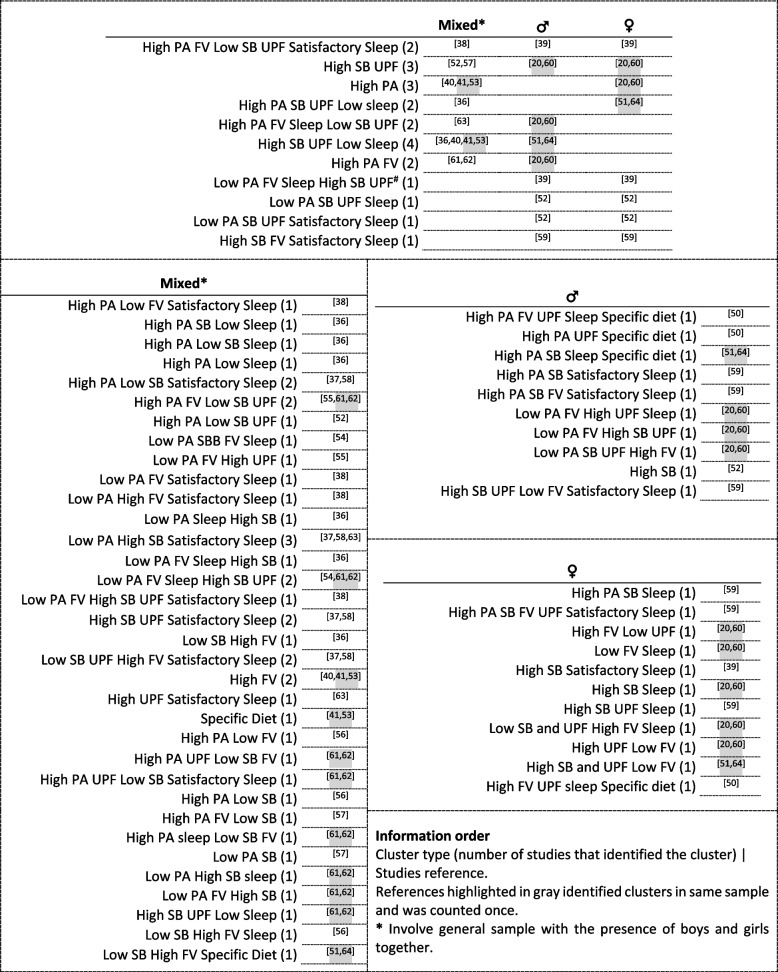
Cluster types (*n* = 66) according to sample stratum and respective studies references. PA: physical activity. SB: sedentary behavior. UPF: ultra-processed foods. FV: fruit and vegetables. Specific Diet” involve consumption of foods that do not frame on FV and UPF (e.g., milk and meat consumption). ^#^ The cluster is also characterized by high discrepancy and quality of sleep

Information about the types of cluster identified from this review are presented in Fig. 3 and in Supplementary material Table S7. Sixty-six mixed cluster types, characterized by both healthy and unhealthy behaviors were identified. More than two thirds of the clusters were identified by one study (*n* = 53). Thirty-four clusters were identified in mixed-sex samples only and the most frequent clusters were “High SB UPF Low Sleep” (*n* = 4), “Low PA High SB Satisfactory Sleep” (*n* = 3), and “High PA” (*n* = 3). Ten clusters’ types were identified in boys and eleven in girls and each profile were identified by one study. The biggest difference found was that most of the girls’ cluster types were characterized by high sleep time, whereas for boys, clusters tended to be characterized by high PA.

#### Indicators associated with cluster types

Of the 23 studies included in this review, 16 examined the relationship between cluster types and health indicators. Adiposity indicators were the most investigated (*n* = 13 studies) [38, 39, 49, 51, 53–56, 58, 59, 61, 63]. Other indicators were investigated to a lesser extent: systolic and diastolic blood pressure [60], insulin resistance [40], quality of life [36, 61], cholesterol and triglycerides [41], and mental health (depression, hyperactivity/inattention, emotional and relationship problems, phobia, anxiety) [50, 51]. In general, null associations were found between cluster types and all health indicators (see Supplementary Material Table S8). However, both positive and negative associations were observed, as shown in Table 2 and below.

**Table 2:** Positive and negative associations between types of clusters and health indicators with their respective study references (*n* = 16)

Indicators	High PA	High PA SB Satisfactory Sleep	High PA SB UPF Low sleep	High PA Sleep Low SB FV	High PA FV UPF Sleep Specific diet	High PA UPF Low SB Satisfactory Sleep	High PA UPF Specific diet	Low PA SB UPF High FV	Low PA SB Satisfactory Sleep	Low PA High SB Satisfactory Sleep	Low PA High SB Sleep	Low PA FV Sleep	Low PA FV Sleep High SB UPF*	Low SB High FV	Low SB High FV Specific diet	Low SB High FV Sleep	Low SB UPF High FV Sleep	High SB FV Satisfactory Sleep	High SB UPF Low Sleep	Specific diet	High UPF Low FV	High UPF Satisfactory Sleep	High FV UPF Sleep Specific diet
**Adiposity variables**																							
Trunk fat	(-) [53]																						
Fat Mass	(-) [53]								(+)♀♂ [58]											(-) [53]			
BMI	(-) [53]								(+)♀♂ [58]		(-) [61]					(-) [56]			(+) [61]	(-) [53]			
Waist circumference	(-) [53]								(+)♀♂ [58]										(+) [61]	(-) [53]			
Overweight																(-) [56]							
Overweight + Obesity		(-)♂ [59]							(+)♀♂ [58]	(+) [63]			(+)♀ [39]					(-)♀ [59]				(+) [63]	
**Metabolic risk factors**																							
SBP								(-)♂ [60]				(+)♂ [60]									(+)♀ [60]		
DBP																	(-)♀ [60]						
LDL cholesterol	(-) [41]																						
HDL cholesterol																			(-) [41]				
Total/HDL cholesterol ratio	(-) [41]																		(+) [41]				
Total cholesterol	(-) [41]																						
Insulin resistance	(-) [40]																		(+) [40]				
**Socio-emotional**																							
Prosocial Behaviors			(+)♀ [51]												(+)♂ [51]								(+)♀ [50]
Hyperactivity/inattention symptoms					(-)♂ [50]										(-)♂ [51]								
Specific phobia symptoms					(-)♂ [50]																		
Separation anxiety symptoms					(-)♂ [50]																		
Generalized anxiety symptoms					(-)♂ [50]																		
Peer relationship problems																							(-)♀ [50]
Emotional symptoms							(-)♂ [50]																
**Quality of life**																							
Social functioning				(+) [61]		(+) [61]																	
Emotional functioning				(+) [61]																			
Psychosocial functioning				(+) [61]																			
Total Score														(+) [36]									

*PA* Physical activity, *SB* Sedentary behavior, *FV* Fruit and vegetables, *UPF* Ultra-processed food. *The cluster is also characterized by high discrepancy and quality of sleep. When the female and male symbols are not specified, it means that the cluster types were identified in mixed samples (with the presence of both boys and girls). ♂ indicates clusters identified in males. ♀ indicates clusters identified in females. ♀♂ indicates clusters identified in girls and in boys separately. (+) indicates positive association (higher average values or greater exposure). (−) indicates negative association (lower average values or lower exposure). *SBP* Systolic blood pressure, *DBP* Diastolic blood pressure, *HDL* High-density lipoprotein, *LDL* Low-density lipoprotein

#### Adiposity indicators

Adolescents allocated to clusters “Low PA High SB Satisfactory Sleep” and “High UPF Satisfactory Sleep” had a higher odds of obesity compared with their peers in the healthiest cluster (“High PA FV Sleep Low SB UPF”) [63]. In addition, adolescents allocated in the cluster “Low SB High FV Sleep” had lower probability to be overweight at 10–11 years [56]. Positive associations with BMI z-score and waist circumference were found among young people for the "High SB UPF Low Sleep" cluster [61]. Girls in the “Low PA FV Sleep High SB UPF” cluster had higher odds of being overweight/obese compared to girls with “High PA FV Low SB UPF Satisfactory Sleep” [39].

Other studies found negative associations (Table 2); youths in clusters characterized by “Specific diet” [53], “High FV” [53], “Low PA High SB Sleep” [61], “Low SB High FV Sleep” [56], and **“**High PA” [53] had lower body mass index [53, 56, 61], fat mass [53], the sum of skinfold thicknesses [53], waist circumference [53], and trunk fat [53]. A lower frequency of overweight/obesity was found in girls and boys in the “High PA SB Satisfactory Sleep” cluster [59]. One study showed significant differences in weight status and adiposity for both boys’ and girls’ clusters [58], while null associations with overweight/obesity were found [38, 54, 55].

#### Metabolic risk factors

Adolescents in “High SB UPF Low sleep” cluster had higher insulin resistance and in “High PA” cluster had lower insulin resistance [40]. Boys in the “Low PA FV Sleep” cluster had higher systolic blood pressure, and girls in “High UPF Low FV” and “Low SB UPF High FV Sleep” profiles had higher and lower diastolic blood pressure, respectively, compared with their peers in “High SB UPF” cluster [60]. In terms of cholesterol indicators, adolescents in “High SB UPF Low Sleep” cluster had lower HDL cholesterol, and in “High PA” cluster had lower total, HDL and LDL cholesterol [41].

#### Quality of life and Social-emotional indicators

Adolescents in “Low SB High FV” cluster had a better overall quality of life [36, 61] and those in “High PA sleep Low SB FV” cluster had a better quality of life and higher emotional, social, and psychosocial functioning. Positive associations were found with prosocial behaviors for the "Low SB High FV Specific diet" clusters among boys and the among girls [50, 51] in "High FV UPF Sleep Specific diet" [50] and "High PA SB UPF Low sleep" [51] clusters. Negative associations were also observed between the "Low SB High FV Specific diet" cluster and hyperactivity/inattention symptoms among boys [51], and between the "High FV UPF Sleep Specific diet" cluster and the variable peer relationship problems among girls [50]. Other negative associations were also observed, for boys only between the "High PA FV UPF Sleep Specific diet" [50] cluster and the following mental health indicators: specific phobia symptoms, separation anxiety symptoms, generalized anxiety symptoms, hyperactivity-inattention symptoms; and between the cluster "High PA UPF Specific diet" [50] and emotional symptoms.

## Discussion

This study systematically reviewed clustering of 24-h movement behaviors and dietary intake and their relationship with health indicators among youth. Overall, sixty-six cluster types were identified for mixed-sex samples, of which, 10 were for boys, and 11 were for girls. Most clusters types comprised healthy and unhealthy behaviors (i.e., mixed cluster types). The majority were identified in youth from high-income countries. Adiposity indicators were the most commonly investigated health outcome. A few associations between cluster types and health indicators were found.

The presence of mixed behavior profiles with the coexistence of unhealthy and healthy behaviors was frequently observed in this review, corroborating existing literature [5, 7, 12]. For example, previous reviews and studies have found that high levels of PA coexisted with high time spent in SB and high consumption of ultra-processed foods and FV in children and adolescents [5, 12]; low levels practicing PA occur with lower time in SB [5, 12, 14]; and high sleep hours coexisted with low quality of diet and low SB and PA [14]. The compensatory health beliefs theory can explain the coexistence of behaviors, which explains that negative effects of unhealthy behaviors (e.g., consuming sweet beverages) can be compensated or neutralized by a healthy behavior (e.g., doing PA) [65]. In contrast, the problem behavior theory [66] posits that engaging in an unhealthy behavior (e.g., watching excess screen time) increases the likelihood of participating in another unhealthy behavior (e.g., consuming UPF) and vice versa. Both theories may help us to understand why certain behaviors cluster together and may result in the coexistence of healthy and unhealthy behaviors.

The main difference in profiles according to sex was that girls’ clusters were characterized by high sleep duration, whereas boys’ clusters by high PA. Previous literature has also identified the presence of mixed lifestyle profiles in boys and girls [25, 39], and studies have demonstrated that girls consistently sleep longer than boys [67, 68]. A possible explanation can be that sleep characteristics are genetically and environmentally determined, although their respective contributions are unknown [68, 69]. What is known is that this sex difference is usually attributed to external influences, such as light and time exposure via use of the screens, academic obligations, and consumption of healthy foods [68, 70, 71]. Boys being more physical active than girls can be explained by points such as sweat, dirt and unwillingness that doing PA can cause [72, 73]. Moreover, girls tend to spend time in domestic tasks and in social activities such as sitting and talking with friends, compared to boys in tasks that involve body movement [74]. In addition, studies have shown that sleeping more or fewer hours than recommended (9–11 h for those aged 5–13 years, and 8–10 for those aged 14–17 years) is associated with increased depressive symptoms, adiposity, blood pressure, and insulin resistance in children and adolescents [75, 76]. Systematic reviews found that children and adolescents with low sleep and PA, and high SB (unhealthiest behavior combination) had unfavorable adiposity, cardiometabolic and mental health [77, 78]. Thus, intervention strategies focused on multiple behaviors at the same time may be important to improve health risk profiles in youth. Additionally, distinct strategies focusing on improving sleep behavior and PA in girls may be an important next step given our finding of girl-specific clusters characterized by excessive sleep duration (above the recommended levels).

Most studies in this review analyzed adiposity indicators in youth [38, 39, 49, 51, 53–56, 58, 59, 61, 63]. Clusters with unhealthy behaviors (e.g., “High UPF Satisfactory Sleep” [63], “Low PA SB Satisfactory Sleep” [58], “Low PA High SB Satisfactory Sleep” [63]**,** “Low PA FV Sleep High SB UPF” [39], Low PA High SB Sleep [61], and "High SB UPF Low Sleep" [61]) were associated with overweight/obesity and increase in adiposity. Interventions based on traditional theories and methodological approaches focusing on a single risk behavior might be insufficient to address complex diseases such as obesity [79]. Although few studies examined indicators such as blood pressure, insulin resistance, cholesterol levels, and mental health indicators, evidence suggested that clusters characterized by unhealthy combination of 24-h movement behaviors and diet are associated with higher insulin resistance [40], blood pressure [60], and prosocial behavior [50, 51]. However, not all associations observed were in the expected direction. For instance, one study found that those in the “High SB UPF Low Sleep” cluster had lower LDL cholesterol [41]. Another study found higher quality of life in adolescents’ at “Low SB High FV” cluster [36]. These findings suggest that future studies should investigate health indicators other than adiposity, and public policy should develop approaching multicomponent strategies to improve health indicators in youth.

One of the strengths of this study is that it is the first study to systematically review the clustering of 24-h movement behavior and diet in mixed-sex samples, and in boys and girls, separately. Boys and girls presented different clusters of behaviors [14, 24, 25]; therefore, the most important implication of this study is to develop strategies to improve multiple behaviors considering their sex-specific profiles. The search strategies were developed in consultation with topic experts and enabled us to identify many potential studies. We also were able to identify and describe the behavior variables used to determine the clusters and the health indicators associated with clusters. Also, our inclusion criteria and authors’ language expertise permitted us to review studies written in multiple languages.

Caution is warranted when generalizing results: 1) the cluster types were based on the classifications presented by the authors of each paper that met the inclusion criteria. However, during the process of describing the clusters, a sequence of criteria and agreement between the researchers was used to ensure parsimonious information was obtained; 2) the wide range of PA, SB, sleep, and diet outcomes/variables made the synthesis of results challenging; however, extraction of behavior frequency, behavior types, intensity, and volumes provided suitable information for characterizing cluster types; 3) we synthesized the direction of the association rather than the magnitude, which is important to understand certain impact/relevance of such behaviors for health-related variables; 4) most of the studies included in this research did not consider maturation as a confounding factor when analyzing associations between clusters types and BMI/adiposity measures; 5) the frequent high risk of bias found mainly for the instruments for assessing sedentary behavior and sleep suggests caution in interpreting the results. It also indicates the need to advance research validating instruments for these behaviors.

We suggest that future studies assess time spent in different types of screen time (e.g., television, cellphone) and include this information as separate input variables in the data-driven clustering analyses. Previous research has shown that different screen-time behaviors may influence health differently [77, 80]. Most studies reported that the time spent sleeping does not differ between clusters, suggesting that future studies should explore sleep behaviors other than sleep duration, such as variables related to quality of sleep (number of awakenings, wake after sleep onset, and sleep efficiency), and sleep stages, enable a more sensitive panorama. Most studies in this review evaluated diet based on consumption of limited food groups, namely FV and UPF. A more detailed assessment of dietary intake is needed. Future studies would benefit from examining dietary patterns that account for interactions between foods, nutrients, and other bioactive components consumed as part of a whole diet and their potential synergistic effects on health. Studies that utilize longitudinal/prospective study designs and employ causal inference analysis are also needed to identify changes in cluster behaviors and their associated factors, in order to examine the effect of intervention strategies. Future cross-sectional and longitudinal studies are needed to examine the association between the clustering of 24-h movement behaviors and diet and other health indicators in addition to adiposity. Few studies provided sufficient detail regarding the analytic decisions made to determine the optimal number of clusters, and the reliability of the resulting cluster solution was rarely reported. Future interventions should test multiple behaviors strategies to promote children and adolescent’s health.

## Conclusion

In summary, this systematic review addressed the clustering of 24-h movement behaviors and diet in youth, and their associations with diverse health indicators. We found a high prevalence of mixed behavior profiles characterized by the presence of at least one unhealthy behavior. Importantly, these patterns differed by sex, with girls more likely to engage in low PA and sleep above recommended levels, when compared to boys. These findings underscore the need to tailor prevention and intervention strategies to sex-specific behavior patterns. Also, further research is needed to understand the underlying environmental determinants that shape health behavior profiles in boys and girls separately. Clusters characterized by high SB, low PA and UPF were associated with higher adiposity, however, few studies examined health indicators other than adiposity, examined all four behaviors included a prospective study design; these are all areas for ongoing research. Nonetheless, the findings from this review suggest that the co-occurrence of unhealthy behaviors may amplify health risks for youth and the presence of unhealthy behaviors alongside healthy ones highlights the importance of using an integrative approach to understanding how these behaviors collectively influence health.

### Supplementary Information

**Supplementary Material 1.**

## Data Availability

The datasets used and/or analyzed during the current study available from the corresponding author on reasonable request.

## Abbreviations

PA: Physical activity
SB: Sedentary behavior
FV: Fruit and vegetables
UPF: Ultra-processed foods
PRISMA: Preferred Reporting Items for Systematic Reviews and Meta-Analysis
SWiM: Synthesis Without Meta-analysis
EPHPP: Effective Public Health Practice Project
