# Supplementary material for: Clusters of 24-hour movement behavior and diet and their relationship with health indicators among youth: a systematic review
Source: BMC Public Health. 2024 Apr 18;24:1080. doi: 10.1186/s12889-024-18364-6 (PMC11027390; doi:10.1186/s12889-024-18364-6)
Supplement: Supplementary file 1 — Supplementary Material 1. [file 12889_2024_18364_MOESM1_ESM.docx]

**Clusters of diet and 24-hours movement behavior and their relationship with health indicators among youth: a systematic review**

**Table S1.** Prisma Checklist


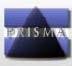
**PRISMA 2020 Checklist**

| **Section and Topic** | **Item #** | **Checklist item** | **Location where item is reported** |
| --- | --- | --- | --- |
| **TITLE** | | |  |
| Title | 1 | Identify the report as a systematic review. | Page 1 |
| **ABSTRACT** | | |  |
| Abstract | 2 | See the PRISMA 2020 for Abstracts checklist. | Page 2 |
| **INTRODUCTION** | | |  |
| Rationale | 3 | Describe the rationale for the review in the context of existing knowledge. | Pages 3, 4 |
| Objectives | 4 | Provide an explicit statement of the objective(s) or question(s) the review addresses. | Page 4 |
| **METHODS** | | |  |
| Eligibility criteria | 5 | Specify the inclusion and exclusion criteria for the review and how studies were grouped for the syntheses. | Pages 5 |
| Information sources | 6 | Specify all databases, registers, websites, organizations, reference lists and other sources searched or consulted to identify studies. Specify the date when each source was last searched or consulted. | Page 5 and Table S1 |
| Search strategy | 7 | Present the full search strategies for all databases, registers and websites, including any filters and limits used. | Table S1 |
| Selection process | 8 | Specify the methods used to decide whether a study met the inclusion criteria of the review, including how many reviewers screened each record and each report retrieved, whether they worked independently, and if applicable, details of automation tools used in the process. | Page 5 and 6 |
| Data collection process | 9 | Specify the methods used to collect data from reports, including how many reviewers collected data from each report, whether they worked independently, any processes for obtaining or confirming data from study investigators, and if applicable, details of automation tools used in the process. | Pages 7, 8 |
| Data items | 10a | List and define all outcomes for which data were sought. Specify whether all results that were compatible with each outcome domain in each study were sought (e.g. for all measures, time points, analyses), and if not, the methods used to decide which results to collect. | Pages 7, 8 |
|  | 10b | List and define all other variables for which data were sought (e.g. participant and intervention characteristics, funding sources). Describe any assumptions made about any missing or unclear information. | Pages 7, 8 |
| Study risk of bias assessment | 11 | Specify the methods used to assess risk of bias in the included studies, including details of the tool(s) used, how many reviewers assessed each study and whether they worked independently, and if applicable, details of automation tools used in the process. | Pages 6, 7 |
| Effect measures | 12 | Specify for each outcome the effect measure(s) (e.g. risk ratio, mean difference) used in the synthesis or presentation of results. | Pages 7, 8 |
| Synthesis methods | 13a | Describe the processes used to decide which studies were eligible for each synthesis (e.g. tabulating the study intervention characteristics and comparing against the planned groups for each synthesis (item #5)). | Pages 7, 8 |
|  | 13b | Describe any methods required to prepare the data for presentation or synthesis, such as handling of missing summary statistics, or data conversions. | Not applicable |
|  | 13c | Describe any methods used to tabulate or visually display results of individual studies and syntheses. | Page 8 |
|  | 13d | Describe any methods used to synthesize results and provide a rationale for the choice(s). If meta-analysis was performed, describe the model(s), method(s) to identify the presence and extent of statistical heterogeneity, and software package(s) used. | Not applicable |
|  | 13e | Describe any methods used to explore possible causes of heterogeneity among study results (e.g. subgroup analysis, meta-regression). | Not applicable |
|  | 13f | Describe any sensitivity analyses conducted to assess robustness of the synthesized results. | Not applicable |
| Reporting bias assessment | 14 | Describe any methods used to assess risk of bias due to missing results in a synthesis (arising from reporting biases). | Pages 6, 7 |
| Certainty assessment | 15 | Describe any methods used to assess certainty (or confidence) in the body of evidence for an outcome. | Not applicable |


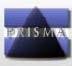
**PRISMA 2020 Checklist**

| **Section and Topic** | **Item #** | **Checklist item** | **Location where item is reported** |
| --- | --- | --- | --- |
| **RESULTS** | | |  |
| Study selection | 16a | Describe the results of the search and selection process, from the number of records identified in the search to the number of studies included in the review, ideally using a flow diagram. | Page 8, 9 and Figure 1 |
|  | 16b | Cite studies that might appear to meet the inclusion criteria, but which were excluded, and explain why they were excluded. | Figure 1 |
| Study characteristics | 17 | Cite each included study and present its characteristics. | Page 9 and Table 1 |
| Risk of bias in studies | 18 | Present assessments of risk of bias for each included study. | Table S2 |
| Results of individual studies | 19 | For all outcomes, present, for each study: (a) summary statistics for each group (where appropriate) and (b) an effect estimates and its precision (e.g. confidence/credible interval), ideally using structured tables or plots. | Pages 10-13, Table 1, 2, Figure 3, Tables S3-S5 |
| Results of syntheses | 20a | For each synthesis, briefly summarize the characteristics and risk of bias among contributing studies. | Page 9-10 and Figure 2 |
|  | 20b | Present results of all statistical syntheses conducted. If meta-analysis was done, present for each the summary estimate and its precision (e.g. confidence/credible interval) and measures of statistical heterogeneity. If comparing groups, describe the direction of the effect. | Not applicable |
|  | 20c | Present results of all investigations of possible causes of heterogeneity among study results. | Not applicable |
|  | 20d | Present results of all sensitivity analyses conducted to assess the robustness of the synthesized results. | Not applicable |
| Reporting biases | 21 | Present assessments of risk of bias due to missing results (arising from reporting biases) for each synthesis assessed. | Not applicable |
| Certainty of evidence | 22 | Present assessments of certainty (or confidence) in the body of evidence for each outcome assessed. | Not applicable |
| **DISCUSSION** | | |  |
| Discussion | 23a | Provide a general interpretation of the results in the context of other evidence. | Pages 13 |
|  | 23b | Discuss any limitations of the evidence included in the review. | Page 16 |
|  | 23c | Discuss any limitations of the review processes used. | Page 16 |
|  | 23d | Discuss implications of the results for practice, policy, and future research. | Page 16-17 |
| **OTHER INFORMATION** | | |  |
| Registration and protocol | 24a | Provide registration information for the review, including register name and registration number, or state that the review was not registered. | Page 4-5 |
|  | 24b | Indicate where the review protocol can be accessed, or state that a protocol was not prepared. | Page 4-5 |
|  | 24c | Describe and explain any amendments to information provided at registration or in the protocol. | Not applicable |
| Support | 25 | Describe sources of financial or non-financial support for the review, and the role of the funders or sponsors in the review. | Page 18 |
| Competing interests | 26 | Declare any competing interests of review authors. | Page 18 |
| Availability of data, code and other materials | 27 | Report which of the following are publicly available and where they can be found: template data collection forms; data extracted from included studies; data used for all analyses; analytic code; any other materials used in the review. | Electronic Supplementary Material |

*From:* Page MJ, McKenzie JE, Bossuyt PM, Boutron I, Hoffmann TC, Mulrow CD, et al. The PRISMA 2020 statement: an updated guideline for reporting systematic reviews. BMJ 2021;372:n71. doi: 10.1136/bmj.n71

For more information, visit: <http://www.prisma-statement.org/>

**Table S2.** SWiM checklist

The citation for the Synthesis Without Meta-analysis explanation and elaboration article is: Campbell M, McKenzie JE, Sowden A, Katikireddi SV, Brennan SE, Ellis S, Hartmann-Boyce J, Ryan R, Shepperd S, Thomas J, Welch V, Thomson H. Synthesis without meta-analysis (SWiM) in systematic reviews: reporting guideline BMJ 2020;368:l6890 <http://dx.doi.org/10.1136/bmj.l6890>

| **SWiM is intended to complement and be used as an extension to PRISMA** | | | |
| --- | --- | --- | --- |
| **SWiM reporting item** | **Item description** | **Page in manuscript where item is reported** | **Other*** |
| *Methods* | | | |
| **1** Grouping studies for synthesis | 1a) Provide a description of, and rationale for, the groups used in the synthesis (e.g., groupings of populations, interventions, outcomes, study design) | Not applicable |  |
|  | 1b) Detail and provide rationale for any changes made subsequent to the protocol in the groups used in the synthesis | Not applicable |  |
| **2** Describe the standardised metric and transformation methods used | Describe the standardised metric for each outcome. Explain why the metric(s) was chosen, and describe any methods used to transform the intervention effects, as reported in the study, to the standardised metric, citing any methodological guidance consulted | Not applicable |  |
| **3** Describe the synthesis methods | Describe and justify the methods used to synthesise the effects for each outcome when it was not possible to undertake a meta-analysis of effect estimates | Not applicable |  |
| **4** Criteria used to prioritise results for summary and synthesis | Where applicable, provide the criteria used, with supporting justification, to select the particular studies, or a particular study, for the main synthesis or to draw conclusions from the synthesis (e.g., based on study design, risk of bias assessments, directness in relation to the review question) | Not applicable |  |
| **SWiM reporting item** | **Item description** | **Page in manuscript where item is reported** | **Other*** |
| **5** Investigation of heterogeneity in reported effects | State the method(s) used to examine heterogeneity in reported effects when it was not possible to undertake a meta-analysis of effect estimates and its extensions to investigate heterogeneity | Not applicable |  |
| **6** Certainty of evidence | Describe the methods used to assess certainty of the synthesis findings | Pages 7, 8 |  |
| **7** Data presentation methods | Describe the graphical and tabular methods used to present the effects (e.g., tables, forest plots, harvest plots).  Specify key study characteristics (e.g., study design, risk of bias) used to order the studies, in the text and any tables or graphs, clearly referencing the studies included | Not applicable |  |
| *Results* | | | |
| **8** Reporting results | For each comparison and outcome, provide a description of the synthesised findings, and the certainty of the findings. Describe the result in language that is consistent with the question the synthesis addresses, and indicate which studies contribute to the synthesis | Pages 8-13 |  |
| *Discussion* |  |  |  |
| **9** Limitations of the synthesis | Report the limitations of the synthesis methods used and/or the groupings used in the synthesis, and how these affect the conclusions that can be drawn in relation to the original review question | Page 16 |  |

PRISMA=Preferred Reporting Items for Systematic Reviews and Meta-Analyses.

*If the information is not provided in the systematic review, give details of where this information is available (e.g., protocol, other published papers (provide citation details), or website (provide the URL)).

**Table S3.** Search strategy

PUBMED

| **Search Group** | **Search Terms** |
| --- | --- |
| Physical Activity | sport* OR sports[mesh] OR sports OR "motor activity"[mesh] OR "motor activity" OR "physical activity" OR "physical activit*" OR exercise[mesh] OR exercise OR "exercise*" OR "physical exercise*" OR "exercise program*" OR "physical education" OR "physical fitness"[mesh] OR "physical fitness" OR "leisure time" OR "leisure activit*" OR "aerobic activity" OR "physical inactivity") |
| Sedentary Behavior | sedentarism OR sedentary OR "sedentary behavior" OR "sedentary behaviors" OR "sedentary behaviour" OR "sedentary behaviours" OR "sedentary lifestyle*" OR "sedentary lifestyle"[mesh] OR "sedentary lifestyle" OR television[mesh] OR television OR "television time" OR "television watch*" OR "TV watch*" OR "screen time" OR "screen viewing" OR "screen media" OR "media screen time" OR "time sitting" OR sitting OR "sitting time" OR computers[mesh] OR computers OR "computer time" OR "computer use" OR "video game*" |
| Diet Behavior | diet[mesh] OR diet OR "diet behavior" OR "diet behaviour" OR "diet consumption" OR "dietary intake" OR "unhealthy diet" OR "healthy diet"[mesh] OR "healthy diet" OR nutrition OR "food behavior" OR "feeding behavior"[mesh] OR "feeding behavior" OR "feeding behaviors" OR "feeding behaviour" OR "feeding behaviours" OR "eating behavior" OR "eating behaviors" OR "eating behaviour" OR "eating behaviours" OR "food consumption" OR "food choice" OR "food intake" OR "food habit" OR "food habits" OR "food preferences"[mesh] OR "food preferences" OR "unhealthy food" OR "nutritional quality" |
| Analysis | "cluster analysis"[mesh] OR "cluster analysis" OR cluster OR cluster* OR clustering OR co-occur OR co-occurrence OR "behavior pattern" OR "behavior patterns" OR "behaviour pattern" OR "behaviour patterns" OR "lifestyle pattern" OR "lifestyle patterns" OR "latent class" OR "factor analysis" OR "factorial analysis" OR simultaneity |
| Population | youth OR adolesce* OR adolescent[mesh] OR adolescent OR adolescent* OR adolescence OR student* OR students[mesh] OR students OR teen* OR teenage* OR schoolchildren OR child* OR child[mesh] OR child OR children[mesh] OR children |

Web of Science

| **Search Group** | **Search Terms** |
| --- | --- |
| Physical Activity | TS=(sport* OR sports OR "motor activity" OR "physical activity" OR "physical activit*" OR exercise OR "exercise*" OR "physical exercise*" OR "exercise program*" OR "physical education" OR "physical fitness" OR "leisure time" OR "leisure activit*" OR "aerobic activity" OR recreation OR "physical inactivity") |
| Sedentary Behavior | TS=(sedentarism OR sedentary OR "sedentary behavior" OR "sedentary behaviors" OR "sedentary behaviour" OR "sedentary behaviours" OR "sedentary lifestyle*" OR "sedentary lifestyle" OR television OR "television time" OR "television watch*" OR "TV watch*" OR "screen time" OR "screen viewing" OR "screen media" OR "media screen time" OR "time sitting" OR sitting OR "sitting time" OR computers OR "computer time" OR "computer use" OR "video game*") |
| Diet Behavior | TS=(diet OR "diet behavior" OR "diet behaviour" OR "diet consumption" OR "dietary intake" OR "unhealthy diet" OR "healthy diet" OR nutrition OR "food behavior" OR "feeding behavior" OR "feeding behaviors" OR "feeding behaviour" OR "feeding behaviours" OR "eating behavior" OR "eating behaviors" OR "eating behaviour" OR "eating behaviours" OR "food consumption" OR "food choice" OR "food intake" OR "food habit" OR "food habits" OR "food preferences" OR "unhealthy food" OR "nutritional quality") |
| Analysis | TS=("cluster analysis" OR cluster OR cluster* OR clustering OR co-occur OR co-occurrence OR "behavior pattern" OR "behavior patterns" OR "behaviour pattern" OR "behaviour patterns" OR "lifestyle pattern" OR "lifestyle patterns" OR "latent class" OR "factor analysis" OR "factorial analysis" OR simultaneity) |
| Population | TS=(youth OR adolesce* OR adolescent OR adolescent* OR adolescence OR student* OR students OR teen* OR teenage* OR schoolchildren OR child* OR child OR children) |

SCOPUS

| **Search Group** | **Search Terms** |
| --- | --- |
| Physical Activity | TITLE-ABS-KEY(sport* OR sports OR "motor activity" OR "physical activity" OR "physical activit*" OR exercise OR "exercise*" OR "physical exercise*" OR "exercise program*" OR "physical education" OR "physical fitness" OR "leisure time" OR "leisure activit*" OR "aerobic activity" OR recreation OR "physical inactivity") |
| Sedentary Behavior | TITLE-ABS-KEY(sedentarism OR sedentary OR "sedentary behavior" OR "sedentary behaviors" OR "sedentary behaviour" OR "sedentary behaviours" OR "sedentary lifestyle*" OR "sedentary lifestyle" OR television OR "television time" OR "television watch*" OR "TV watch*" OR "screen time" OR "screen viewing" OR "screen media" OR "media screen time" OR "time sitting" OR sitting OR "sitting time" OR computers OR "computer time" OR "computer use" OR "video game*") |
| Diet Behavior | TITLE-ABS-KEY(diet OR "diet behavior" OR "diet behaviour" OR "diet consumption" OR "dietary intake" OR "unhealthy diet" OR "healthy diet" OR nutrition OR "food behavior" OR "feeding behavior" OR "feeding behaviors" OR "feeding behaviour" OR "feeding behaviours" OR "eating behavior" OR "eating behaviors" OR "eating behaviour" OR "eating behaviours" OR "food consumption" OR "food choice" OR "food intake" OR "food habit" OR "food habits" OR "food preferences" OR "unhealthy food" OR "nutritional quality") |
| Analysis | TITLE-ABS-KEY("cluster analysis" OR cluster OR cluster* OR clustering OR co-occur OR co-occurrence OR "behavior pattern" OR "behavior patterns" OR "behaviour pattern" OR "behaviour patterns" OR "lifestyle pattern" OR "lifestyle patterns" OR "latent class" OR "factor analysis" OR "factorial analysis" OR simultaneity) |
| Population | TITLE-ABS-KEY(youth OR adolesce* OR adolescent OR adolescent* OR adolescence OR student* OR students OR teen* OR teenage* OR schoolchildren OR child* OR child OR children) |

LILACS, MEDLINE AND PSYCINFO

| **Search Group** | **Search Terms** |
| --- | --- |
| Physical Activity | (sport OR sports OR "motor activity" OR "physical activity" OR "physical activities" OR exercise OR exercises OR "physical exercise" OR "exercise program*" OR "physical education" OR "physical fitness" OR "leisure time" OR "leisure activity" OR "leisure activities" OR "aerobic activity" OR recreation OR "physical inactivity") |
| Sedentary Behavior | (sedentarism OR sedentary OR "sedentary behavior" OR "sedentary behaviors" OR "sedentary behaviour" OR "sedentary behaviours" OR "sedentary lifestyles" OR "sedentary lifestyle" OR television OR "television time" OR "television watch" OR "television watches" OR "TV watch" OR "TV watching" OR "TC watches" OR "screen time" OR "screen viewing" OR "screen media" OR "media screen time" OR "time sitting" OR sitting OR "sitting time" OR computers OR "computer time" OR "computer use" OR "video game" OR "video games") |
| Diet Behavior | (diet OR "diet behavior" OR "diet behaviour" OR "diet consumption" OR "dietary intake" OR "unhealthy diet" OR "healthy diet" OR nutrition OR "food behavior" OR "feeding behavior" OR "feeding behaviors" OR "feeding behaviour" OR "feeding behaviours" OR "eating behavior" OR "eating behaviors" OR "eating behaviour" OR "eating behaviours" OR "food consumption" OR "food choice" OR "food intake" OR "food habit" OR "food habits" OR "food preferences" OR "unhealthy food" OR "nutritional quality") |
| Clustering | ("cluster analysis" OR cluster OR cluster* OR clustering OR co-occur OR co-occurrence OR "behavior pattern" OR "behavior patterns" OR "behaviour pattern" OR "behaviour patterns" OR "lifestyle pattern" OR "lifestyle patterns" OR "latent class" OR "factor analysis" OR "factorial analysis" OR simultaneity) |
| Population | (youth OR adolesce* OR adolescent OR adolescent* OR adolescence OR student* OR students OR teen* OR teenage* OR schoolchildren OR child* OR child OR children) |

**Table S4.** Assessment of the bias risk of studies.

| **Article (publication year)** | **Selection bias** | | **Study design** | | | | **Assessment tool** | | | | | | | | | | | | **Withdrawals and drop-outs** | | |
| --- | --- | --- | --- | --- | --- | --- | --- | --- | --- | --- | --- | --- | --- | --- | --- | --- | --- | --- | --- | --- | --- |
|  |  |  |  |  |  |  | **PA** | | | **Diet** | | | **SB** | | | **Sleep** | | |  |  |  |
|  | **Q1** | **Bias** | **Q2** | **Q3** | **Q4** | **Bias** | **Q5** | **Q6** | **Bias** | **Q5** | **Q6** | **Bias** | **Q5** | **Q6** | **Bias** | **Q5** | **Q6** | **Bias** | **Q7** | **Q8** | **Bias** |
| Androutsos (2014)^1^ ^a^ | 0 | Moderate | 1 | 1 | 1 | Strong | 0 | 0 | Weak | 1 | 1 | Strong | 0 | 1 | Weak | 0 | 1 | Weak | 1 | 1 | Strong |
| Collese (2018)^2^ ^b^ | -1 | Weak | 1 | 1 | 1 | Strong | 1 | 1 | Strong | 1 | 1 | Strong | 1 | 1 | Strong | 1 | 1 | Strong | 0 | 0/? | Weak |
| Descarpentrie (2021)^3^ | 1 | Strong | 1 | 1 | 1 | Strong | 0 | 1 | Weak | 1 | 1 | Strong | 0 | 1 | Weak | 0 | 1 | Weak | 1 | -1 | Weak |
| Descarpentrie (2022)^4^ | 1 | Strong | 1 | 1 | 1 | Strong | 0 | 1 | Weak | 0 | 1 | Weak | 1 | 1 | Strong | 0 | 1 | Weak | 1 | 1 | Strong |
| Descarpentrie (2023)^5^ | 1 | Strong | 1 | 1 | 1 | Strong | 0 | 1 | Weak | 1 | 1 | Strong | 0 | 1 | Weak | 0 | 1 | Weak | 1 | -1 | Weak |
| D’ Souza (2021)^6^ | -1 | Weak | 1 | 1 | 1 | Strong | 1 | 1 | Strong | 1 | 1 | Strong | 1 | 1 | Strong | 1 | 1 | Strong | 1 | -1 | Weak |
| D’ Souza (2022)^7^ | -1 | Weak | 1 | 1 | 1 | Strong | 1 | 1 | Strong | 1 | 1 | Strong | 1 | 1 | Strong | 1 | 1 | Strong | 1 | -1 | Weak |
| Dumuid (2017)^8^ | 0 | Moderate | 0 | 1 | * | Weak | 1 | 1 | Strong | 1 | 1 | Strong | 0 | 1 | Weak | 1 | 1 | Strong | 1 | 1 | Strong |
| Dumuid (2017)^9^ ^c^ | -1 | Weak | 0 | 1 | 1 | Moderate | 1 | 1 | Strong | 1 | 1 | Strong | 0 | 1 | Weak | 1 | 1 | Strong | 1 | -1 | Weak |
| Dumuid (2016)^10^ ^c^ | 1 | Strong | 1 | 1 | 1 | Strong | 1 | 1 | Strong | 1 | 1 | Strong | 1 | 1 | Strong | 1 | 1 | Strong | 1 | 0 | Moderate |
| Fernández-Alvira (2013)^11^ ^a^ | 0 | Moderate | 1 | 1 | 1 | Strong | 0 | 1 | Weak | 0 | 1 | Weak | 0 | 1 | Weak | 1 | 1 | Strong | 0 | 1 | Strong |
| Ferrar and Golley (2015)^12^ ^a^ | c | Strong | 0 | -1 | 1 | Weak | 1 | 1 | Strong | 0 | 1 | Weak | 1 | 1 | Strong | 0 | 1 | Weak | 0 | 1 | Strong |
| Knebel (2022)^13^ | 1 | Strong | 1 | 1 | 1 | Strong | 1 | 1 | Strong | 1 | 1 | Strong | 1 | 1 | Strong | 1 | 1 | Strong | 1 | 0 | Moderate |
| Magee (2013)^14^ ^a^ | ? | Weak | 0 | -1 | 1 | Weak | 0 | 1 | Weak | 0 | 1 | Weak | 0 | 1 | Weak | 0 | 1 | Weak | 0 | ? | Weak |
| Miguel-Berges (2017)^15^ ^a^ | 0 | Moderate | 0 | 1 | 1 | Moderate | 1 | 1 | Strong | 1 | 1 | Strong | 1 | 1 | Strong | 1 | 1 | Strong | 1 | 0 | Moderate |
| Moraes (2016)^16^ ^c^ | -1 | Weak | 1 | 1 | 1 | Strong | 1 | 1 | Strong | 1 | 1 | Strong | 1 | 1 | Strong | 1 | 1 | Strong | 1 | 0 | Moderate |
| Moschonis (2012)^17^ ^a^ | 0 | Moderate | 1 | 1 | 1 | Strong | 1 | 1 | Strong | 1 | 1 | Strong | 1 | 1 | Strong | 0 | 1 | Weak | 1 | 0 | Moderate |
| Moschonis (2013)^18^ ^a^ | 0 | Moderate | 1 | 1 | 1 | Strong | 1 | 1 | Strong | 1 | 1 | Strong | 1 | 1 | Strong | 0 | 1 | Weak | 1 | 0 | Moderate |
| Nuutinen (2017)^19^ ^a^ | 1 | Strong | 1 | 1 | 1 | Strong | 1 | 1 | Strong | 1 | 1 | Strong | 1 | 1 | Strong | 1 | 1 | Strong | 0 | 1 | Strong |
| Pereira (2015)^20^ ^a^ | 1 | Strong | 1 | 1 | 1 | Strong | 1 | 1 | Strong | 1 | 1 | Strong | 1 | 1 | Strong | 1 | 1 | Strong | 0 | ? | Weak |
| Pérez-Rodrigo (2015)^21^ ^a^ | 1 | Strong | 1 | 1 | 1 | Strong | 1 | 1 | Strong | 1 | 1 | Strong | 1 | 1 | Strong | 0 | 1 | Weak | 1 | -1 | Weak |
| Saldanha-Gomes (2020)^22^ | 1 | Strong | 1 | 1 | 1 | Strong | 0 | 1 | Weak | 1 | 1 | Strong | 0 | 1 | Weak | 0 | 1 | Weak | 0 | -1 | Weak |
| Wiersma (2022)^23^ | 1 | Strong | 1 | 1 | 1 | Strong | 1 | 1 | Strong | 1 | 1 | Strong | 0 | 1 | Weak | 0 | 1 | Weak | 0 | 1 | Strong |

PA: physical activity; SB: sedentary behavior; Q1: Are the individuals selected to participate in the study likely to be representative of the target population?; Q2: Is there a description of the representativeness of the sample?; Q3: Was the sampling method described?; Q4: Was the method appropriate?; Q5: Is there a prior validation report of the tool?; Q6: Is there information that makes it possible to replicate the tool?; Q7: Were withdrawals and drop-outs reported in terms of numbers and/or reasons per group?; Q8: Indicate the percentage of participants completing the study; ?: impossible to determine; *The selection method differed among study countries. ^a^ High-Income Countries; ^b^ Upper-Middle Income Countries; ^c^ Involves samples from more than one country and with different income classification (Intercontinental Studies with Different Income Countries).

**Table S5.** Instruments used and questionnaires classification according to each behavior.

| **Author(s) (publication year)** | **Instruments classification** | | | |
| --- | --- | --- | --- | --- |
|  | **PA** | **SB** | **Sleep** | **Diet** |
| Androutsos (2014)^1^ | Undefined | Undefined | Undefined-reproductible | Interview (24-h recall 2-week days plus one weekend day) |
| Collese (2018)^2^ | Defined | Defined | Defined | Defined |
| Descarpentrie (2021)^3^ ^#^ | Undefined-reproductible | Undefined-reproductible | Undefined-reproductible | Defined |
| Descarpentrie (2022)^4^ | Undefined-reproductible | Undefined-reproductible | Undefined-reproductible | Undefined-reproductible |
| Descarpentrie (2023)^5^ ^#^ | Undefined-reproductible | Undefined-reproductible | Undefined-reproductible | Defined |
| D’ Souza (2021)^6^ | Accelerometer and questionnaire (Defined) | Accelerometer and questionnaire (Defined) | Defined | Defined |
| D’ Souza (2022)^7^ | Accelerometer and questionnaire (Defined) | Accelerometer and questionnaire (Defined) | Defined | Defined |
| Dumuid (2017)^8^ | Accelerometer (Defined) | Accelerometer (Defined) | Accelerometer (Defined) | Defined |
| Dumuid (2017)^9^ | Accelerometer (Defined) | Undefined | Accelerometer (Defined) | Defined |
| Dumuid (2016)^10^ | Accelerometer (Defined) | Undefined | Accelerometer (Defined) | Defined |
| Fernández-Alvira (2013)^11^ | Defined | Defined | Defined | Defined |
| Ferrar and Golley (2015)^12^ | Defined | Defined | Defined | Defined |
| Knebel (2022)^13^ | Defined | Defined | Defined | Defined |
| Magee (2013)^14^ | Dieries | Dieries | Diarie | Undefined-reproductible |
| Miguel-Berges (2017)^15^ | Pedometer (Defined)* | Defined | Defined | Defined |
| Moraes (2016)^16^ | Defined | Defined | Defined | Defined |
| Moschonis (2012)^17^ | Defined | 24-h recall | Undefined-reproductible | Interview (24-h recall 2-week days plus one weekend day) |
| Moschonis (2013)^18^ | Defined | 24-h recall | Undefined-reproductible | Interview (24-h recall 2-week days plus one weekend day) |
| Nuutinen (2017)^19^ | Defined | Defined | Defined | Defined |
| Pereira (2015)^20^ | Accelerometer (Defined) | Defined | Accelerometer (Defined) | Defined |
| Pérez-Rodrigo (2015)^21^ | Defined | Defined | Undefined-reproductible | Interview 24-h recall (one day) plus three-day record |
| Saldanha-Gomes (2020)^22^ | Undefined-reproductible | Undefined-reproductible | Undefined-reproductible | Defined |
| Wiersma (2022)^23^ | Accelerometer (Defined) and questionnaire (Undefined-reproductible) | Accelerometer (Defined) and questionnaire (Undefined-reproductible) | Undefined-reproductible | Defined |

PA: physical activity. SB: sedentary behavior. * Used two instruments (pedometer and questionnaire). ^#^Interview. (1) Defined (reported the validation process); (2) Undefined (reported question and/or response option and instrument reference); (3) Undefined-Reproducible (reported question and response options but no instrument reference)

**Table S6.** Behavioral outcomes used to define PA, sedentary behavior, sleep and diet included in data driven cluster procedures.

|  | | **Physical activity outcomes** | | | | | | | | | | | | | | | | | | | | | | | | | | | | | | | | | | | | | | | | | |
| --- | --- | --- | --- | --- | --- | --- | --- | --- | --- | --- | --- | --- | --- | --- | --- | --- | --- | --- | --- | --- | --- | --- | --- | --- | --- | --- | --- | --- | --- | --- | --- | --- | --- | --- | --- | --- | --- | --- | --- | --- | --- | --- | --- |
| **Author (publication year)** | | LTPA | | Total PA (min/day) | | Overall, PA (3 domains) (min/day) | | | Overall, PA (3 domains) (min/week) | | Overall, PA (2 domains) (min/week) | | LPA (min/day) | | MPA (min/day) | | VPA (min/day) | | MVPA (min/day) | | Play (min/day) | | Team sports (min/day) | | Non-team sports (min/day) | | Active transport (min/day) | | | Organized sport/PA or active play (h/day) | | Organized sports (min/day) | | Sports (hour/week) | | Biking (min/day) | Walking (min/day) | Outdoor play (days/week) | Outdoor play (min/day) | Outdoor play (hour/day) | Walking (types of transport) | Walking (min/day) | Walking (hour/day) |
| Androutsos (2014)^1^ | |  | |  | |  | | |  | |  | |  | |  | |  | |  | |  | |  | |  | |  | | |  | |  | |  | |  |  |  |  |  |  |  |  |
| Collese (2018)^2^ | |  | |  | |  | | |  | |  | |  | |  | |  | |  | |  | |  | |  | |  | | |  | |  | |  | |  |  |  |  |  |  |  |  |
| Descarpentrie (2021)^3#^ | |  | |  | |  | | |  | |  | |  | |  | |  | |  | |  | |  | |  | |  | | |  | |  | |  | |  |  |  |  |  |  |  |  |
| Descarpentrie (2022)^4^ | |  | |  | |  | | |  | |  | |  | |  | |  | |  | |  | |  | |  | |  | | |  | |  | |  | |  |  |  |  |  |  |  |  |
| Descarpentrie (2023)^5#^ | |  | |  | |  | | |  | |  | |  | |  | |  | |  | |  | |  | |  | |  | | |  | |  | |  | |  |  |  |  |  |  |  |  |
| D’ Souza (2021)^6^ | |  | |  | |  | | |  | |  | |  | |  | |  | |  | |  | |  | |  | |  | | |  | |  | |  | |  |  |  |  |  |  |  |  |
| D’ Souza (2022)^7^ | |  | |  | |  | | |  | |  | |  | |  | |  | |  | |  | |  | |  | |  | | |  | |  | |  | |  |  |  |  |  |  |  |  |
| Dumuid (2017)^8^ | |  | |  | |  | | |  | |  | |  | |  | |  | |  | |  | |  | |  | |  | | |  | |  | |  | |  |  |  |  |  |  |  |  |
| Dumuid (2017)^9^ | |  | |  | |  | | |  | |  | |  | |  | |  | |  | |  | |  | |  | |  | | |  | |  | |  | |  |  |  |  |  |  |  |  |
| Dumuid (2016)^10^ | |  | |  | |  | | |  | |  | |  | |  | |  | |  | |  | |  | |  | |  | | |  | |  | |  | |  |  |  |  |  |  |  |  |
| Fernández-Alvira (2013)^11^ | |  | |  | |  | | |  | |  | |  | |  | |  | |  | |  | |  | |  | |  | | |  | |  | |  | |  |  |  |  |  |  |  |  |
| Ferrar and Golley (2015)^12^ | |  | |  | |  | | |  | |  | |  | |  | |  | |  | |  | |  | |  | |  | | |  | |  | |  | |  |  |  |  |  |  |  |  |
| Knebel (2022)^13^ | |  | |  | |  | | |  | |  | |  | |  | |  | |  | |  | |  | |  | |  | | |  | |  | |  | |  |  |  |  |  |  |  |  |
| Magee (2013)^14^ | |  | |  | |  | | |  | |  | |  | |  | |  | |  | |  | |  | |  | |  | | |  | |  | |  | |  |  |  |  |  |  |  |  |
| Miguel-Berges (2017)^15^ | |  | |  | |  | | |  | |  | |  | |  | |  | |  | |  | |  | |  | |  | | |  | |  | |  | |  |  |  |  |  |  |  |  |
| Moraes (2016)^16^ | |  | |  | |  | | |  | |  | |  | |  | |  | |  | |  | |  | |  | |  | | |  | |  | |  | |  |  |  |  |  |  |  |  |
| Moschonis (2012)^17^ | |  | |  | |  | | |  | |  | |  | |  | |  | |  | |  | |  | |  | |  | | |  | |  | |  | |  |  |  |  |  |  |  |  |
| Moschonis (2013)^18^ | |  | |  | |  | | |  | |  | |  | |  | |  | |  | |  | |  | |  | |  | | |  | |  | |  | |  |  |  |  |  |  |  |  |
| Nuutinen (2017)^19^ | |  | |  | |  | | |  | |  | |  | |  | |  | |  | |  | |  | |  | |  | | |  | |  | |  | |  |  |  |  |  |  |  |  |
| Pereira (2015)^20^ | |  | |  | |  | | |  | |  | |  | |  | |  | |  | |  | |  | |  | |  | | |  | |  | |  | |  |  |  |  |  |  |  |  |
| Pérez-Rodrigo (2015)^21^ | |  | |  | |  | | |  | |  | |  | |  | |  | |  | |  | |  | |  | |  | | |  | |  | |  | |  |  |  |  |  |  |  |  |
| Wiersma (2022)^23^ | |  | |  | |  | | |  | |  | |  | |  | |  | |  | |  | |  | |  | |  | | |  | |  | |  | |  |  |  |  |  |  |  |  |
| Note: LTPA: leisure time physical activity. LPA: light physical activity. MVPA: moderate vigorous physical activity. | | | | | | | | | | | | | | | | | | | | | | | | | | | | | | | | | | | | | | | | | | | |
|  | | **Sedentary behavior outcomes** | | | | | | | | | | | | | | | | | | | | | | | | | | | | | | | | | |  |  |  |  |  |  |  |  |
| **Author (publication year)** | | Screen time: TV+PC+VG (hours/day) | | Hours/day - screen time (TV+PC) | | Min/day of screen time (TV+PC+VG) | | Screen time: TV+PC (min/day) | Sedentary time - accelerometry (min/day) | | TV (min/day) | | PC (min/day) | | VG (min/day) | | TV (h/day) | | PC (h/day) | | VG (h/day) | | Cellphone/Smartphone | | Social interaction (min/day) | | Eating (min/day) | | Quiet time (min/day) | Study/homework/music (min/day) | | Reading (min/day) | | Passive transport (min/day) | |  |  |  |  |  |  |  |  |
| Androutsos (2014)^1^ | |  | |  | |  | |  |  | |  | |  | |  | |  | |  | |  | |  | |  | |  | |  |  | |  | |  | |  |  |  |  |  |  |  |  |
| Collese (2018)^2^ | |  | |  | |  | |  |  | |  | |  | |  | |  | |  | |  | |  | |  | |  | |  |  | |  | |  | |  |  |  |  |  |  |  |  |
| Descarpentrie (2021)^3#^ | |  | |  | |  | |  |  | |  | |  | |  | |  | |  | |  | |  | |  | |  | |  |  | |  | |  | |  |  |  |  |  |  |  |  |
| Descarpentrie (2022)^4^ | |  | |  | |  | |  |  | |  | |  | |  | |  | |  | |  | |  | |  | |  | |  |  | |  | |  | |  |  |  |  |  |  |  |  |
| Descarpentrie (2023)^5#^ | |  | |  | |  | |  |  | |  | |  | |  | |  | |  | |  | |  | |  | |  | |  |  | |  | |  | |  |  |  |  |  |  |  |  |
| D’ Souza (2021)^6^ | |  | |  | |  | |  |  | |  | |  | |  | |  | |  | |  | |  | |  | |  | |  |  | |  | |  | |  |  |  |  |  |  |  |  |
| D’ Souza (2022)^7^ | |  | |  | |  | |  |  | |  | |  | |  | |  | |  | |  | |  | |  | |  | |  |  | |  | |  | |  |  |  |  |  |  |  |  |
| Dumuid (2017)^8^ | |  | |  | |  | |  |  | |  | |  | |  | |  | |  | |  | |  | |  | |  | |  |  | |  | |  | |  |  |  |  |  |  |  |  |
| Dumuid (2017)^9^ | |  | |  | |  | |  |  | |  | |  | |  | |  | |  | |  | |  | |  | |  | |  |  | |  | |  | |  |  |  |  |  |  |  |  |
| Dumuid (2016)^10^ | |  | |  | |  | |  |  | |  | |  | |  | |  | |  | |  | |  | |  | |  | |  |  | |  | |  | |  |  |  |  |  |  |  |  |
| Fernández-Alvira (2013)^11^ | |  | |  | |  | |  |  | |  | |  | |  | |  | |  | |  | |  | |  | |  | |  |  | |  | |  | |  |  |  |  |  |  |  |  |
| Ferrar and Golley (2015)^12^ | |  | |  | |  | |  |  | |  | |  | |  | |  | |  | |  | |  | |  | |  | |  |  | |  | |  | |  |  |  |  |  |  |  |  |
| Knebel (2022)^13^ | |  | |  | |  | |  |  | |  | |  | |  | |  | |  | |  | |  | |  | |  | |  |  | |  | |  | |  |  |  |  |  |  |  |  |
| Magee (2013)^14^ | |  | |  | |  | |  |  | |  | |  | |  | |  | |  | |  | |  | |  | |  | |  |  | |  | |  | |  |  |  |  |  |  |  |  |
| Miguel-Berges (2017)^15^ | |  | |  | |  | |  |  | |  | |  | |  | |  | |  | |  | |  | |  | |  | |  |  | |  | |  | |  |  |  |  |  |  |  |  |
| Moraes (2016)^16^ | |  | |  | |  | |  |  | |  | |  | |  | |  | |  | |  | |  | |  | |  | |  |  | |  | |  | |  |  |  |  |  |  |  |  |
| Moschonis (2012)^17^ | |  | |  | |  | |  |  | |  | |  | |  | |  | |  | |  | |  | |  | |  | |  |  | |  | |  | |  |  |  |  |  |  |  |  |
| Moschonis (2013)^18^ | |  | |  | |  | |  |  | |  | |  | |  | |  | |  | |  | |  | |  | |  | |  |  | |  | |  | |  |  |  |  |  |  |  |  |
| Nuutinen (2017)^19^ | |  | |  | |  | |  |  | |  | |  | |  | |  | |  | |  | |  | |  | |  | |  |  | |  | |  | |  |  |  |  |  |  |  |  |
| Pereira (2015)^20^ | |  | |  | |  | |  |  | |  | |  | |  | |  | |  | |  | |  | |  | |  | |  |  | |  | |  | |  |  |  |  |  |  |  |  |
| Pérez-Rodrigo (2015)^21^ | |  | |  | |  | |  |  | |  | |  | |  | |  | |  | |  | |  | |  | |  | |  |  | |  | |  | |  |  |  |  |  |  |  |  |
| Wiersma (2022)^23^ | |  | |  | |  | |  |  | |  | |  | |  | |  | |  | |  | |  | |  | |  | |  |  | |  | |  | |  |  |  |  |  |  |  |  |
| Note: Acc: accelerometer. TV: television. PC: computer. VG: video game. | | | | | | | | | | | | | | | | | | | | | | | | | | | | | | | | | | | |  |  |  |  |  |  |  |  |

|  | **Sleep outcomes** | | | | | |
| --- | --- | --- | --- | --- | --- | --- |
| **Author (publication year)** | Hours/day | Min/day | Accelerometer (min/day) | Accelerometer (hour/day) | Sleep Discrepancy | Sleep quality |
| Androutsos (2014)^1^ |  |  |  |  |  |  |
| Collese (2018)^2^ |  |  |  |  |  |  |
| Descarpentrie (2021)^3#^ |  |  |  |  |  |  |
| Descarpentrie (2022)^4^ |  |  |  |  |  |  |
| Descarpentrie (2023)^5#^ |  |  |  |  |  |  |
| D’ Souza (2021)^6^ |  |  |  |  |  |  |
| D’ Souza (2022)^7^ |  |  |  |  |  |  |
| Dumuid (2017)^8^ |  |  |  |  |  |  |
| Dumuid (2017)^9^ |  |  |  |  |  |  |
| Dumuid (2016)^10^ |  |  |  |  |  |  |
| Fernández-Alvira (2013)^11^ |  |  |  |  |  |  |
| Ferrar and Golley (2015)^12^ |  |  |  |  |  |  |
| Knebel (2022)^13^ |  |  |  |  |  |  |
| Magee (2013)^14^ |  |  |  |  |  |  |
| Miguel-Berges (2017)^15^ |  |  |  |  |  |  |
| Moraes (2016)^16^ |  |  |  |  |  |  |
| Moschonis (2012)^17^ |  |  |  |  |  |  |
| Moschonis (2013)^18^ |  |  |  |  |  |  |
| Nuutinen (2017)^19^ |  |  |  |  |  |  |
| Pereira (2015)^20^ |  |  |  |  |  |  |
| Pérez-Rodrigo (2015)^21^ |  |  |  |  |  |  |
| Wiersma (2022)^23^ |  |  |  |  |  |  |

|  | **Diet outcomes** | | | | | | | | | | | | | | | | | | | | | | | | | | | | | | | |
| --- | --- | --- | --- | --- | --- | --- | --- | --- | --- | --- | --- | --- | --- | --- | --- | --- | --- | --- | --- | --- | --- | --- | --- | --- | --- | --- | --- | --- | --- | --- | --- | --- |
| **Author (publication year)** | F&V (portion/day) | F&V (CDC daily recommendation) | F&V (times/day) | F&V (serves/week) | F&V (times/week) | Water (portion/day) | Sugary drinks (portion/day) | Sugary drinks (grams/day) | Sugary drinks (times/day) | Sweet beverages (portion/day) | Sweet beverages (ml/day) | Sweet beverages (serves/week) | Sweet beverages (times/week) | Sweet snacks and desserts (time/week) | Rice/pasta (times/week) | Bread (times/week) | Crisps (times/week) | French fries (times/week) | Savory discretionary food intake (times/day) | Sweet discretionary food intake (times/day) | Fast food (times/day) | Junk food (times/week) | Score - positive food group | Score - negative food group | PCA - F&V | PCA - Healthy eating pattern# | PCA - Unhealthy eating pattern* | PCA - ultra processed foods | EFA - Mediterranean | EFA- Sandwich | EFA - Pasta | EFA - Milk-sugary foods |
| Androutsos (2014)^1^ |  |  |  |  |  |  |  |  |  |  |  |  |  |  |  |  |  |  |  |  |  |  |  |  |  |  |  |  |  |  |  |  |
| Collese (2018)^2^ |  |  |  |  |  |  |  |  |  |  |  |  |  |  |  |  |  |  |  |  |  |  |  |  |  |  |  |  |  |  |  |  |
| Descarpentrie (2021)^3#^ |  |  |  |  |  |  |  |  |  |  |  |  |  |  |  |  |  |  |  |  |  |  |  |  |  |  |  |  |  |  |  |  |
| Descarpentrie (2022)^4^ |  |  |  |  |  |  |  |  |  |  |  |  |  |  |  |  |  |  |  |  |  |  |  |  |  |  |  |  |  |  |  |  |
| Descarpentrie (2023)^5#^ |  |  |  |  |  |  |  |  |  |  |  |  |  |  |  |  |  |  |  |  |  |  |  |  |  |  |  |  |  |  |  |  |
| D’ Souza (2021)^6 £^ |  |  |  |  |  |  |  |  |  |  |  |  |  |  |  |  |  |  |  |  |  |  |  |  |  |  |  |  |  |  |  |  |
| D’ Souza (2022)^7 £^ |  |  |  |  |  |  |  |  |  |  |  |  |  |  |  |  |  |  |  |  |  |  |  |  |  |  |  |  |  |  |  |  |
| Dumuid (2017)^8^ |  |  |  |  |  |  |  |  |  |  |  |  |  |  |  |  |  |  |  |  |  |  |  |  |  |  |  |  |  |  |  |  |
| Dumuid (2017)^9^ |  |  |  |  |  |  |  |  |  |  |  |  |  |  |  |  |  |  |  |  |  |  |  |  |  |  |  |  |  |  |  |  |
| Dumuid (2016)^10^ |  |  |  |  |  |  |  |  |  |  |  |  |  |  |  |  |  |  |  |  |  |  |  |  |  |  |  |  |  |  |  |  |
| Fernández-Alvira (2013)^11^ |  |  |  |  |  |  |  |  |  |  |  |  |  |  |  |  |  |  |  |  |  |  |  |  |  |  |  |  |  |  |  |  |
| Ferrar and Golley (2015)^12^ |  |  |  |  |  |  |  |  |  |  |  |  |  |  |  |  |  |  |  |  |  |  |  |  |  |  |  |  |  |  |  |  |
| Knebel (2022)^13^ |  |  |  |  |  |  |  |  |  |  |  |  |  |  |  |  |  |  |  |  |  |  |  |  |  |  |  |  |  |  |  |  |
| Magee (2013)^14^ |  |  |  |  |  |  |  |  |  |  |  |  |  |  |  |  |  |  |  |  |  |  |  |  |  |  |  |  |  |  |  |  |
| Miguel-Berges (2017)^15^ |  |  |  |  |  |  |  |  |  |  |  |  |  |  |  |  |  |  |  |  |  |  |  |  |  |  |  |  |  |  |  |  |
| Moraes (2016)^16^ |  |  |  |  |  |  |  |  |  |  |  |  |  |  |  |  |  |  |  |  |  |  |  |  |  |  |  |  |  |  |  |  |
| Moschonis (2012)^17^ |  |  |  |  |  |  |  |  |  |  |  |  |  |  |  |  |  |  |  |  |  |  |  |  |  |  |  |  |  |  |  |  |
| Moschonis (2013)^18^ |  |  |  |  |  |  |  |  |  |  |  |  |  |  |  |  |  |  |  |  |  |  |  |  |  |  |  |  |  |  |  |  |
| Nuutinen (2017)^19^ |  |  |  |  |  |  |  |  |  |  |  |  |  |  |  |  |  |  |  |  |  |  |  |  |  |  |  |  |  |  |  |  |
| Pereira (2015)^20^ |  |  |  |  |  |  |  |  |  |  |  |  |  |  |  |  |  |  |  |  |  |  |  |  |  |  |  |  |  |  |  |  |
| Pérez-Rodrigo (2015)^21^ |  |  |  |  |  |  |  |  |  |  |  |  |  |  |  |  |  |  |  |  |  |  |  |  |  |  |  |  |  |  |  |  |
| Wiersma (2022)^23^ |  |  |  |  |  |  |  |  |  |  |  |  |  |  |  |  |  |  |  |  |  |  |  |  |  |  |  |  |  |  |  |  |

|  | **Diet outcomes - continued** | | | | | | | | | | | | | | | | | | | | | | | | | |
| --- | --- | --- | --- | --- | --- | --- | --- | --- | --- | --- | --- | --- | --- | --- | --- | --- | --- | --- | --- | --- | --- | --- | --- | --- | --- | --- |
| **Author (publication year)** | Breakfast and meal frequency | Fruit (serves/day) | Fruit (grams/day) | Fruit (times/day) | Fruit (times/week) | Fruit (not specified) | Vegetable (serves/day) | Vegetable (grams/day) | Vegetables (times/day) | Vegetable (times/week) | Vegetable (not specified) | Whole-grain products (serves/day) | Whole-grain products (grams/day) | Processed meat (times/week) | Red meat (serves/day) | Red meat (grams/day) | Red meat (not specified) | Fish (grams/day) | Fish (times/week) | Fish (not specified) | Yogurts (times/week) | Dairy (serves/day) | Dairy (grams/day) | Dairy (times/week) | Healthy fat (serves/day) | Extra foods (serves/day) |
| Androutsos (2014)^1^ |  |  |  |  |  |  |  |  |  |  |  |  |  |  |  |  |  |  |  |  |  |  |  |  |  |  |
| Collese (2018)^2^ |  |  |  |  |  |  |  |  |  |  |  |  |  |  |  |  |  |  |  |  |  |  |  |  |  |  |
| Descarpentrie (2021)^3#^ |  |  |  |  |  |  |  |  |  |  |  |  |  |  |  |  |  |  |  |  |  |  |  |  |  |  |
| Descarpentrie (2022)^4^ |  |  |  |  |  |  |  |  |  |  |  |  |  |  |  |  |  |  |  |  |  |  |  |  |  |  |
| Descarpentrie (2023)^5#^ |  |  |  |  |  |  |  |  |  |  |  |  |  |  |  |  |  |  |  |  |  |  |  |  |  |  |
| D’ Souza (2021)^6 £^ |  |  |  |  |  |  |  |  |  |  |  |  |  |  |  |  |  |  |  |  |  |  |  |  |  |  |
| D’ Souza (2022)^7 £^ |  |  |  |  |  |  |  |  |  |  |  |  |  |  |  |  |  |  |  |  |  |  |  |  |  |  |
| Dumuid (2017)^8^ |  |  |  |  |  |  |  |  |  |  |  |  |  |  |  |  |  |  |  |  |  |  |  |  |  |  |
| Dumuid (2017)^9^ |  |  |  |  |  |  |  |  |  |  |  |  |  |  |  |  |  |  |  |  |  |  |  |  |  |  |
| Dumuid (2016)^10^ |  |  |  |  |  |  |  |  |  |  |  |  |  |  |  |  |  |  |  |  |  |  |  |  |  |  |
| Fernández-Alvira (2013)^11^ |  |  |  |  |  |  |  |  |  |  |  |  |  |  |  |  |  |  |  |  |  |  |  |  |  |  |
| Ferrar and Golley (2015)^12^ |  |  |  |  |  |  |  |  |  |  |  |  |  |  |  |  |  |  |  |  |  |  |  |  |  |  |
| Knebel (2022)^13^ |  |  |  |  |  |  |  |  |  |  |  |  |  |  |  |  |  |  |  |  |  |  |  |  |  |  |
| Magee (2013)^14^ |  |  |  |  |  |  |  |  |  |  |  |  |  |  |  |  |  |  |  |  |  |  |  |  |  |  |
| Miguel-Berges (2017)^15^ |  |  |  |  |  |  |  |  |  |  |  |  |  |  |  |  |  |  |  |  |  |  |  |  |  |  |
| Moraes (2016)^16^ |  |  |  |  |  |  |  |  |  |  |  |  |  |  |  |  |  |  |  |  |  |  |  |  |  |  |
| Moschonis (2012)^17^ |  |  |  |  |  |  |  |  |  |  |  |  |  |  |  |  |  |  |  |  |  |  |  |  |  |  |
| Moschonis (2013)^18^ |  |  |  |  |  |  |  |  |  |  |  |  |  |  |  |  |  |  |  |  |  |  |  |  |  |  |
| Nuutinen (2017)^19^ |  |  |  |  |  |  |  |  |  |  |  |  |  |  |  |  |  |  |  |  |  |  |  |  |  |  |
| Pereira (2015)^20^ |  |  |  |  |  |  |  |  |  |  |  |  |  |  |  |  |  |  |  |  |  |  |  |  |  |  |
| Pérez-Rodrigo (2015)^21^ |  |  |  |  |  |  |  |  |  |  |  |  |  |  |  |  |  |  |  |  |  |  |  |  |  |  |
| Wiersma (2022)^23^ |  |  |  |  |  |  |  |  |  |  |  |  |  |  |  |  |  |  |  |  |  |  |  |  |  |  |

**Table S7.** Clusters Detail

| **Authors (publication year)** | **Clusters types identified in paper (N / %)** | **Cluster types defined by review authors** |
| --- | --- | --- |
| Androutsos (2014)^1^ | Component 1  Component 2  Component 3 Component 4 Component 5 | Component 1 – High FV Component 2 – High FV Component 3 – High SB UPF Low sleep Component 4 – High PA Component 5 – Specific diet |
| Collese (2018)^2^ | **HELENA Boys (n=592)** Cluster 1 – Sedentary (n=141, 23.9%) Cluster 2 – Healthy (n=253, 42.7%) Cluster 3 – Unhealthy Eating (n=197, 33.3%) **HELENA Girls (n=660)** Cluster 1 – Sedentary (n=137, 20.8%) Cluster 2 – Active (n=105, 15.9%) Cluster 3 – Unhealthy Eating (n=183, 27.7%) Cluster 4 – Healthy eating (n=237, 35.9%) **BRACAH Boys (n=312)**  Cluster 1 – Sedentary (n=139, 44.7%)  Cluster 2 – Active (n=67, 21.5%) Cluster 3 – Healthy Eating (n=105, 33.8%)  **BRACAH Girls (n=370)** Cluster 1 – Sedentary (n=66, 17.8%) Cluster 2 – Active (n= 54, 14.5%) Cluster 3 – Unhealthy eating (n=134, 36.2%) Cluster 4 – Healthy Eating (n=116, 31.5%) | **HELENA Boys** Cluster 1 – High SB UPF Cluster 2 – High PA FV Sleep Low SB UPF  Cluster 3 – Low PA FV Sleep **HELENA Girls** Cluster 1 – High SB UPF  Cluster 2 – High PA  Cluster 3 – Low FV Sleep Cluster 4 – Low SB UPF High FV Sleep **BRACAH Boys**  Cluster 1 – Low PA FV High SB UPF Cluster 2 – High PA FV Cluster 3 – Low PA SB UPF High FV  **BRACAH Girls** Cluster 1 – High SB Sleep Cluster 2 – High PA  Cluster 3 – High UPF Low FV Cluster 4 – High FV Low UPF |
| Descarpentrie (2021)^3^ | **Boys (n=519)** Component 1 – Unhealthy Component 2 – Healthy  Component 3 – Mixed **Girls (n=459)** Component 1 – Unhealthy Component 2 – Healthy  Component 3 – Mixed | **Boys (n=519)** Component 1 – High SB UPF Low sleep Component 2 – Low SB High FV Specific diet Component 3 – High PA SB sleep Specific diet **Girls (n=459)** Component 1 – High SB UPF Low FV Component 2 – Low SB High FV Specific diet Component 3 – High PA SB UPF Low sleep |
| Descarpentrie (2022)^4^ | **Boys (n=121)** Component 1 – LP1 Component 2 – LP2 **Girls (n=114)** Component 1 – LP1 | **Boys (n=121)** Component 1 – High PA FV UPF Sleep Specific diet Component 2 – High PA UPF Specific diet  **Girls (n=114)** Component 1 – High FV UPF sleep Specific diet |
| Descarpentrie (2023)^5^ | **Boys (n=519)** Component 1 – Unhealthy Component 2 – Healthy  Component 3 – Mixed **Girls (n=459)** Component 1 – Unhealthy Component 2 – Healthy  Component 3 – Mixed | **Boys (n=519)** Component 1 – High SB UPF Low sleep Component 2 – Low SB High FV Specific diet Component 3 – High PA SB sleep Specific diet **Girls (n=459)** Component 1 – High SB UPF Low FV Component 2 – Low SB High FV Specific diet Component 3 – High PA SB UPF Low sleep |
| D’ Souza (2021)^6^ | **K-means**  1 – Unhealthy (n=133) 2 – Active healthy eaters (n=102)  3 – Active sleepers, non-sedentary unhealthy eaters (n=197) **LPA** 1 – Unhealthy (n=206) 2 – Active healthy eaters (n=84) 3 – Active non-sedentary unhealth eaters (n=142)  **PCA** 1 – Component 1 – Active sleepers, non-sedentary unhealth eaters 2 – Component 2 – Activate healthy eaters  3 – Component 3 – Poor sleepers and sedentary snackers  4 – Component 3 – Inactive sedentary sleepers | **K-means**  1 – Low PA FV sleep High SB UPF 2 – High PA FV Low SB UPF  3 – High PA sleep Low SB FV **LPA** 1 – Low PA FV High SB 2 – High PA FV 3 – High PA UPF Low SB FV **PCA** 1 – High PA UPF Low SB Satisfactory Sleep  2 – High PA FV 3 – High SB UPF Low Sleep  4 – Low PA High SB sleep |
| D’ Souza (2022)^7^ | **K-means**  1 – Unhealthy (n=133) 2 – Active healthy eaters (n=102)  3 – Active sleepers, non-sedentary unhealthy eaters (n=197) **LPA** 1 – Unhealthy (n=206) 2 – Active healthy eaters (n=84) 3 – Active non-sedentary unhealth eaters (n=142)  **PCA** 1 – Component 1 – Active sleepers, non-sedentary unhealth eaters 2 – Component 2 – Activate healthy eaters  3 – Component 3 – Poor sleepers and sedentary snackers  4 – Component 3 – Inactive sedentary sleepers | **K-means**  1 – Low PA FV sleep High SB UPF 2 – High PA FV Low SB UPF  3 – High PA sleep and Low SB FV **LPA** 1 – Low PA FV High SB 2 – High PA FV 3 – High PA UPF Low SB FV **PCA** 1 – High PA UPF Low SB Satisfactory Sleep  2 –High PA FV 3 – High SB UPF Low Sleep  4 – Low PA High SB sleep |
| Dumuid (2017)^8^ | **Australia:** Sitters (n=105, 24%); Actives (n=98,23%);Junk food screeners (n=99, 23%); All-rounders (n=127, 30%). **Brazil:** Retro-actives (n=134, 31%); Sitters (n=127, 29%); Junk food techno-actives (n=56, 13%), Techno-active (n=118, 27%). **Canada:** Junk food screeners (n=152, 31%); Junk food techno-actives (n=22, 4%); Sitters (n=136, 27%); All-rounders (n=185, 37%). **China:** Junk food screeners (n=47, 10%); All-rounders (n=104, 23%); Actives (n=167, 36%); Sitters (n=140, 31%). **Colombia:** Low sleep (n=244, 30%); Sitters (n=161, 20%); Junk food techno-actives (n=180, 22%), All-rounders (n=235, 29%). **England:** Junk food screeners (n=94, 25%); Actives (n=87, 23%); Sitters (n=84, 23%); All-rounders (n=108, 29%). **Finland:** Actives (n=150, 35%); All-rounders (n=122, 28%); Sitters (n=139, 32%); Junk food screeners (n=21, 5%). **India:** All-rounders (n=119, 23%); Sitters (n=18, 35%), Junk food screeners (n=59, 13%); Actives (n=165, 31%). **Kenya:** Retro-active (n=123, 27%), Lightly active (n=130, 29%); Junk food techno-actives (n=98, 22%); Sitters (n=99, 22%). **Portugal:** All-rounders (n=164,29%); Actives (n=166, 30%); Sitters (n=158, 28%); Junk food screeners (n=74 , 13%). **South Africa:** Low food intake (n=99, 27%); Sitters (n=92, 25%); Retro-actives (n=81, 23%); Junk food screeners (n=89, 25%). **US:** Sitters (n=88, 21%); Actives (n=113, 27%); All-rounders (n=150, 36%); Junk food screeners (n=67; 16%). | Sitters – Low PA Sleep High SB Actives – High PA Low Sleep  Junk food screeners – High SB UPF Low Sleep Junk food techno actives – High PA SB UPF Low Sleep Techno-actives – High PA SB Low Sleep Retro-actives – High PA Low SB Sleep All-rounders – Low SB High FV  Low food intake – Low PA FV Sleep High SB  Lightly active – Moderate PA Low sleep – High PA SB Low sleep |
| Dumuid (2017)^9^ | Junk Food Screeners (n=19, 6.7%) All-Rounders (n=30, 10.7%) Actives (n=24, 8.5%) Sitters (n=27, 9.5%) | Junk Food Screeners – High SB UPF Low FV Satisfactory Sleep All-Rounders – Low SB UPF High FV Satisfactory Sleep Actives – High PA Low SB Satisfactory Sleep Sitters – Low PA High SB Satisfactory Sleep |
| Dumuind (2016)^10^ | **Boys** (n = 2576)  Cluster 1 – Junk Food Screeners (n=274, 9%)  Cluster 2 – Actives (n=887, 34%)  Cluster 3 – Sitters (n=702, 27%)  Cluster 4 – All-Rounders (n=713, 287%)  **Girls** (n = 3134)  Cluster 1 – Junk Food Screeners (n=325, 10%)  Cluster 2 – Actives (n=958, 30%)  Cluster 3 – Sitters (n=991, 32%)  Cluster 4 – All-Rounders (n=860, 28%) | **Boys**   Cluster 1 – High SB UPF Satisfactory Sleep Cluster 2 – High PA Low SB Satisfactory Sleep  Cluster 3 – Low PA Low SB Satisfactory Sleep  Cluster 4 – Low SB High FV Satisfactory Sleep  **Girls**   Cluster 1 – High SB UPF Satisfactory Sleep Cluster 2 – High PA Low SB Satisfactory Sleep  Cluster 3 – Low PA Low SB Satisfactory Sleep  Cluster 4 – Low SB High FV Satisfactory Sleep |
| Fernández-Alvira (2013)^11^ | **Girls (n= 2871)** Active pattern (n=641, 22.3%) Long sleepers inactive pattern (n=615, 21.4%) Sedentary sugared drinks consumers (n=436, 15.2%) Short sleepers inactive pattern (n=529, 18.4%) Low activity (n=650, 22.6%) **Boys (n=2413)** Active pattern (n=540, 22.4%) Long sleepers inactive pattern (n=479, 19.9%) Sedentary sugared drinks consumers (n=240, 9.9%) Short sleepers inactive pattern (n=753, 31.2%) Sedentary pattern (n=401, 16.6%) | **Girls** Active pattern – High PA Low SB UPF Long sleepers’ inactive pattern – High Sleep Low PA SB UPF Satisfactory Sleep Sedentary sugared drinks consumers – High SB UPF Short sleepers’ inactive pattern – Low Sleep PA SB UPF  Low activity – Low PA SB UPF Satisfactory Sleep **Boys** Active pattern – High PA Low SB UPF Long sleepers’ inactive pattern – Low PA SB UPF Satisfactory Sleep Sedentary sugared drinks consumers – High SB UPF  Short sleepers inactive pattern – Low Sleep PA SB UPF  Sedentary pattern – High SB |
| Ferrar (2015)^12^ | **Boys (n = 930)** Healthy Academic (n=189, 20.3%) Active sitter (n=318, 34.2%) Unhealthy (n=328, 35.3%) Social Helper (n=95, 10.2%) **Girls (n = 923)** Unhealthy Screener (n=309, 33.5%) Healthy Academic (n=257, 27.8%) Healthy and Unhealthy (n=213, 23.1%) Active Sitter (n=144, 15.6%). | **Boys**  Healthy Academic – High PA SB FV Satisfactory Sleep Active sitter – High PA SB Satisfactory Sleep Unhealthy – High SB UPF Low FV Satisfactory Sleep  Social Helper – High SB FV Satisfactory Sleep **Girls** Unhealthy Screener – High SB UPF Sleep  Healthy Academic – High SB FV Satisfactory Sleep Healthy and Unhealthy – High PA SB FV UPF Satisfactory Sleep Active Sitter – High PA SB Sleep |
| Knebel (2022)^13^ | Phubbers (n=379, 50.53%) Healthier (n=200, 26.67%) Gamers (n=171, 22.80%) | Phubbers – Low PA SB  Healthier – High PA FV Low SB Gamers – High SB UPF |
| Magee (2013)^14^ | Cluster 1 healthy (n=508, 27,7%)  Cluster 2 sedentary (n=455, 24,8%)  Cluster 3 unhealthy eaters (n=870, 47,5%) | Cluster 1 – High PA FV S**leep** Low SB UPF Cluster 2 – Low PA High SB Satisfactory Sleep  Cluster 3 – High UPF Satisfactory Sleep |
| Miguel-Berges (2017)^15^ | Cluster 1: Healthy diet and low activity (NR)  Cluster 2: Active (NR)  Cluster 3: Healthy lifestyle (NR)  Cluster 4: High water and screen time; low fruit and vegetables (NR) Cluster 5: Unhealthy lifestyle (NR)  Cluster 6: High fruit and vegetables consumers (NR) | Cluster 1 – Low PA High FV Satisfactory Sleep Cluster 2 – High PA Low FV Satisfactory Sleep Cluster 3 – High PA FV Low SB UPF Satisfactory Sleep Cluster 4 – Low PA FV High SB Satisfactory Sleep Cluster 5 – Low PA FV High SB UPF Satisfactory Sleep Cluster 6 – Low PA High FV Satisfactory Sleep |
| Moraes (2016)^16^ | **HELENA Boys (n=592)** Cluster 1 – Sedentary (n=141, 23.9%) Cluster 2 – Healthy (n=253, 42.7%) Cluster 3 – Unhealthy Eating (n=197, 33.3%) **HELENA Girls (n=660)** Cluster 1 – Sedentary (n=137, 20.8%) Cluster 2 – Active (n=105, 15.9%) Cluster 3 – Unhealthy Eating (n=183, 27.7%) Cluster 4 – Healthy eating (n=237, 35.9%) **BRACAH Boys (n=312)**  Cluster 1 – Sedentary (n=139, 44.7%)  Cluster 2 – Active (n=67, 21.5%) Cluster 3 – Healthy Eating (n=105, 33.8%)  **BRACAH Girls (n=370)** Cluster 1 – Sedentary (n=66, 17.8%) Cluster 2 – Active (n= 54, 14.5%) Cluster 3 – Unhealthy eating (n=134, 36.2%) Cluster 4 – Healthy Eating (n=116, 31.5%) | **HELENA Boys** Cluster 1 – High SB UPF Cluster 2 – High PA FV Sleep Low SB UPF  Cluster 3 – Low PA FV Sleep **HELENA Girls** Cluster 1 – High SB UPF  Cluster 2 – High PA  Cluster 3 – Low FV Sleep Cluster 4 – Low SB UPF High FV Sleep **BRACAH Boys**  Cluster 1 – Low PA FV High SB UPF Cluster 2 – High PA FV Cluster 3 – Low PA SB UPF High FV  **BRACAH Girls** Cluster 1 – High SB Sleep Cluster 2 – High PA  Cluster 3 – High UPF Low FV Cluster 4 – High FV Low UPF |
| Moschonis (2012)^17^ | Component 1  Component 2  Component 3  Component 4  Component 5 | Component 1 – Specific diet  Component 2 – High FV  Component 3 – High SB UPF Low Sleep  Component 4 – High PA  Component 5 – Specific diet |
| Moschonis (2013)^18^ | Component 1  Component 2  Component 3  Component 4  Component 5 | Component 1 – Specific diet  Component 2 – High FV  Component 3 – High SB UPF Low Sleep  Component 4 – High PA  Component 5 – Specific diet |
| Nuutinen (2017)^19^ | **Boys**  Cluster 1 – Health lifestyle (n=996, 55%)  Cluster 2 – High screen time, unhealthy lifestyle (n=308, 17%) Cluster 3 – Low/moderate screen time, unhealthy lifestyle (n=510, 28%) **Girls** Cluster 1 – Health lifestyle (n=1112, 54%)  Cluster 2 – High screen time, unhealthy lifestyle (n=505, 25%) Cluster 3 – Poor sleep, unhealthy lifestyle (n=434, 21%) | **Boys** Cluster 1 – High PA FV Low SB UPF Satisfactory Sleep duration, (High quality and Low sleep discrepancy) Cluster 2 – Low PA FV Sleep High SB UPF (High discrepancy of sleep)  Cluster 3 – Low PA FV Sleep High SB UPF (High discrepancy and quality of sleep) **Girls**  Cluster 1 – High PA FV Low SB UPF Satisfactory Sleep duration, (High quality and Low sleep discrepancy) Cluster 2 – Low PA FV Sleep High SB UPF (Low discrepancy and quality)  Cluster 3 – Low SB Satisfactory Sleep (Low discrepancy and quality of sleep) |
| Pereira (2015)^20^ | Class 1 – Sedentary, Poorer Diet Quality (n=242, 35.3%) Class 2 – Insufficiently Active, better diet quality (n=444, 64.7%) | Class 1 – Low PA FV Sleep High SB UPF  Class 2 – Low PA UPF FV Sleep |
| Pérez-Rodrigo (2016)^21^ | 1. Unhealthier Lifestyle Pattern (n=319, 76.9%) 2. Healthier Lifestyle Pattern (n=96, 23.1%) | 1. Low PA FV High UPF 2. High PA FV Low SB UPF |
| Wiersma (2022)^23^ | Component 1 – High activity  Component 2 – Low screen time, High sleep and Health diet  Component 3 – High outdoor play | Component 1 – High PA Low SB  Component 2 – Low SB High FV Sleep  Component 3 – High PA Low FV |
| Note. PA: Physical activity. SB: Sedentary behavior. FV: Fruit and vegetables. UPF: ultra-processed foods. Specific Diet” involve consumption of foods that do not frame on FV and UPF (e.g., milk and meat consumption). | | |

**Table S8.** Association between cluster types and health indicators (n = 16).

| **Authors (publication year)** | **Association Analysis** | **Indicators associated with clusters** | **Direction of association** |
| --- | --- | --- | --- |
| Androutsos (2014)^1^ | Linear regression | Insulin Resistance (continuous) | High FV **(0)** High FV **(0)** High SB UPF Low sleep **(+)** (β = 0.043/ p = 0.040) High PA **(-)** (β = -0.061/ p = 0.003) Specific diet **(0)** |
| Descarpentrie (2022)^4^ | Linear regression | Specific phobia symptoms (continuous)  Separation anxiety symptoms (continuous)  Generalized anxiety symptoms (continuous)  Depression/dysthymia symptoms (continuous)  Opposition symptoms (continuous)  Conduct problem symptoms (continuous)  Hyperactivity–inattention symptoms (continuous)  Strength and competencies (continuous)  Emotional symptoms (continuous)  Peer relationship problems (continuous)  Conduct problem symptoms (continuous)  Prosocial behaviors (continuous) | **Boys**  High PA FV UPF Sleep Specific diet: Specific phobia symptoms (-) (β = -0.20 [95%CI = -0.39; -0.01]); Separation anxiety symptoms (-) (β = -0.22 [95%CI = -0.37; -0.06); Generalized anxiety symptoms (-) (β = -0.21 [95%CI = -0.39; -0.04]); Hyperactivity-inattention symptoms (-) (β = -0.20 [95%CI = -0.34; -0,06)  High PA UPF Specific diet: Emotional symptoms (-) (β = -0.32 [95%CI = -0.50; -0.14])  **Girls**  High FV UPF Sleep Specific diet: Peer relationship problems (-) (β = -0.24 [95%CI = -0.40; -0.09]); Prosocial behaviors (+) (β = 0.31 [95%CI = 0.17; 0.45]) |
| Descarpentrie (2023)^5^ | Linear regression | Prosocial behaviors (continuous)  Total difficulties (continuous)  Hyperactivity/inattention symptoms (continuous)  Conduct problems (continuous)  Emotional problems (continuous)  Peer relationship problems (continuous)  BMI z-score | **Boys**  High SB UPF Low sleep **(0)**  Low SB High FV Specific diet: Prosocial behaviors (+) (β = 0.14 [95%CI = 0.01; 0.26]); Hyperactivity/inattention symptoms (-) (β = -0.12 [95%CI = -0.12])  High PA SB sleep Specific diet **(0)**  **Girls**  High SB UPF Low FV **(0)**  Low SB High FV Specific diet **(0)**  High PA SB UPF Low sleep: Prosocial behaviors (+) (β = 0.12 [95%CI = 0.01; 0.24] |
| D’Souza (2022)^7^ | Linear regression | BMI z-score (continuous)  Waist circumference (continuous)  Health-related quality of life (HRQoL) | **CA**  High PA sleep Low SB FV: emotional functioning HRQoL (+) (β = 6.02 / p-value = 0.021); social functioning HRQoL (+) (β = 7.50 / p-value = 0.017); psychosocial functioning HRQoL (+) (β = 5.70 / p-value = 0.035)  **PCA**  High PA UPF Low SB Satisfactory Sleep: Social functioning (+) (β = 1.80 / p-value = 0.035)  High PA FV **(0)**  High SB UPF Low Sleep: BMI z-score (+) (β = 0.09 / p-value = 0.015); Waist circumference (+) (β = 0.07 / p-value = 0.028)  Low PA High SB Sleep: BMI z-score (-) (β = -0.10 / p-value = 0.021) |
| Dumuid (2017)^8^ | ANCOVA | Health-related quality of life (HRQoL) (continuous) | Low PA Sleep High SB High PA Low Sleep  High SB UPF Low Sleep High PA SB UPF Low Sleep High PA SB Low Sleep High PA Low SB Sleep Low SB High FV **(greatest HRQoL compared to others)** Low PA FV Sleep High SB  High PA SB Low sleep |
| Dumuind (2016)^10^ | ANCOVA | WC, body fat, BMI z-score, percentage of overweight/obesity (continuous) | **Boys**   High SB UPF Satisfactory Sleep High PA Low SB Satisfactory Sleep  Low PA SB Satisfactory Sleep **(greatest WC, body fat, BMI z-score, and percentage of overweight/obesity compared to others)**  Low SB High FV Satisfactory Sleep  **Girls**  High SB UPF Satisfactory Sleep High PA Low SB Satisfactory Sleep  Low PA SB Satisfactory Sleep **(greatest WC, body fat, BMI z-score, and percentage of overweight/obesity compared to others)**  Low SB High FV Satisfactory Sleep |
| Ferrar (2015)^12^ | χ2 | Weight status (binary – overweight/obese versus non-overweight/obese) | **Boys**  High PA SB FV Satisfactory Sleep High PA SB Satisfactory Sleep **(Lower frequency of overweight/obesity)** High SB UPF Low FV Satisfactory Sleep  High SB FV Satisfactory Sleep **Girls** High SB UPF Sleep  High SB FV Satisfactory Sleep **(Lower frequency of overweight/obesity)** High PA SB FV UPF Satisfactory Sleep High PA SB Sleep |
| Magee (2013)^14^ | Logistic regression | Obesity (classified by obesity task force - overweight/obesity) | High PA FV Sleep Low SB UPF **(ref.)** Low PA High SB Satisfactory Sleep **(+)** (OR = 1.61 [95%CI = 1.16; 2.22] baseline / OR = 1.59 [95%CI = 1.06; 2.38] follow-up) High UPF Satisfactory Sleep **(0/+)** (0 baseline / OR = 1.47 [95%CI = 1.03; 2.13] follow-up) |
| Miguel-Berges (2017)^15^ | Chi-square | BMI categories (normal weight, overweight, and obesity) | Low PA High FV Satisfactory Sleep (0) High PA Low FV Satisfactory Sleep (0) High PA FV Low SB UPF Satisfactory Sleep (0) Low PA FV High SB Satisfactory Sleep (0) Low PA FV High SB UPF Satisfactory Sleep (0) Low PA High FV Satisfactory Sleep (0) |
| Moraes (2016)^16^ | Multilevel linear regression | Systolic blood pressure (SBP) (continuous)  Diastolic blood pressure (DBP) (continuous) | **HELENA Boys** High SB UPF **(ref)** High PA FV Sleep Low SB UPF **(0)**  Low PA FV Sleep **(+)** (SBP: β = 4.10 [95%CI = 0.80; 7.40]) **HELENA Girls** High SB UPF **(ref)** High PA **(0)**  Low FV Sleep **(0)** Low SB UPF High FV Sleep **(-)** (DBP: β = −2.46 [95%CI = -4.62; -0.30]) **BRACAH Boys**  Low PA FV High SB UPF **(ref)** High PA FV **(0)** Low PA SB UPF High FV **(-)** (SBP: β = −2.79 [95%CI = -3.10; -0.15])  **BRACAH Girls** High SB Sleep **(ref)** High PA **(0)**  High UPF Low FV **(+)** (SBP: β = 4.54 [95%CI = 1.29; 7.79]) High FV Low UPF **(0)** |
| Moschonis (2012)^17^ | Linear regression | Total cholesterol, HDL, LDL, Triglycerides (continuous) | Specific diet **(0)** High FV **(0)** High SB UPF Low Sleep **(-/+)**: HDL cholesterol (β = −0.077; p-value = <0.001); Total/HDL cholesterol ratio (β = 0.049; p-value = 0.025) High PA **(-):** total cholesterol (β = −0.064; p-value = 0.006); LDL (β = −0.065; p-value = 0.004); Total/HDL cholesterol ratio (β = −0.043; p-value = 0.049) Specific diet **(0)** |
| Moschonis (2013)^18^ | Linear regression | BMI, WC, sum of skinfold thicknesses (SST), fat mass, trunk fat, visceral trunk fat (continuous) | Specific diet **(-)**: BMI (β = −0.06; p-value = 0.007); WC (β = −0.06; p-value = 0.007); SST (β = −0.08; p-value = <0.001); fat mass (β = −0.05; p-value = 0.029) High FV **(-)**: SST (β = −0.07; p-value = 0.002) High SB UPF Low Sleep **(0)** High PA **(-)**: BMI (β = −0.05; p-value = 0.024); WC (β = −0.06; p-value = 0.012); fat mass (β −0.08; p-value = <0.001); trunk fat (β = −0.09; p-value = 0.002) Specific diet (0) |
| Nuutinen (2017)^19^ | Logistic regression | Overweight including obesity (binary - overweight/obesity and normal, classified by Cole) | **Boys** High PA FV Low SB UPF Satisfactory Sleep duration, (High quality and Low sleep discrepancy) **(ref.)** Low PA FV Sleep High SB UPF (High sleep discrepancy) **(0)** Low PA FV Sleep High UPF SB (High sleep discrepancy and quality) **(0)** **Girls**  High PA FV Low SB UPF Satisfactory Sleep duration, (High quality and Low sleep discrepancy) **(ref.)** Low PA FV Sleep duration High SB UPF (High sleep discrepancy and quality) **(+) (β 1.42)** Low SB Satisfactory Sleep (Low sleep discrepancy and quality) **(0)** |
| Pereira (2015)^20^ | Pearson x² | Overweight including obesity (binary - overweight/obesity and normal, classified by WHO) | Low PA FV Sleep High SB UPF **(0)**  Low PA UPF FV Sleep **(0)** |
| Pérez-Rodrigo (2016)^21^ | Pearson x² | Overweight including obesity (binary - overweight/obesity and normal, classified by Cole) | Low PA FV High UPF **(0)** High PA FV Low SB UPF **(0)** |
| Wiesman (2022)^23^ | Logistic regression  Linear regression | Overweight (yes/no)  BMI z-score | High PA Low SB **(0)**  Low SB High FV Sleep: Overweight (lower probability to be overweight at 10-11 years; OR = 0.776 [95%CI = 0.66; 0.92]); BMI z-score (-) (β = -0.071 [95%CI = -0.11; -0.03).  High PA Low FV **(0)** |

ANCOVA: Analysis of Covariance WC: weight circumference. BMI: body mass index. PA: physical activity. SB: sedentary behavior. UPF: ultra-processed foods. FV: fruit and vegetables. Specific Diet” involve consumption of foods that do not frame on FV and UPF (e.g., milk and meat consumption); (+) indicates positive association; (-) indicates negative association; (0) indicates no association.

**REFERENCES**

1. Androutsos O, Moschonis G, Mavrogianni C, Roma-Giannikou E, Chrousos GP, Kanaka-Gantenbein C, et al. Identification of lifestyle patterns, including sleep deprivation, associated with insulin resistance in children: the Healthy Growth Study. Eur J Clin Nutr. março de 2014;68(3):344–9.

2. Collese TS, De Moraes ACF, Fernández-Alvira JM, Michels N, De Henauw S, Manios Y, et al. How do energy balance-related behaviors cluster in adolescents? Int J Public Health. março de 2019;64(2):195–208.

3. Descarpentrie A, Saldanha-Gomes C, Guivarch C, Dargent-Molina P, De Lauzon-Guillain B, Plancoulaine S, et al. Family Socioecological Correlates of Lifestyle Patterns in Early Childhood: A Cross-Sectional Study from the EDEN Mother–Child Cohort. Nutrients. 26 de outubro de 2021;13(11):3803.

4. Descarpentrie A, Estevez M, Brabant G, Vandentorren S, Lioret S. Lifestyle Patterns of Children Experiencing Homelessness: Family Socio-Ecological Correlates and Links with Physical and Mental Health. Int J Environ Res Public Health. 5 de dezembro de 2022;19(23):16276.

5. Descarpentrie A, Bernard JY, Vandentorren S, Melchior M, Galéra C, Chia A, et al. Prospective associations of lifestyle patterns in early childhood with socio‐emotional and behavioural development. Paediatr Perinat Epidemiol. janeiro de 2023;37(1):69–80.

6. D’Souza NJ, Downing K, Abbott G, Orellana L, Lioret S, Campbell KJ, et al. A comparison of children’s diet and movement behaviour patterns derived from three unsupervised multivariate methods. Scott JA, organizador. PLOS ONE. 27 de julho de 2021;16(7):e0255203.

7. D’Souza NJ, Zheng M, Abbott G, Lioret S, Hesketh KD. Differing associations with childhood outcomes using behavioural patterns derived from three data reduction techniques. Int J Epidemiol. 19 de abril de 2022;52(2):577–88.

8. Dumuid D, Olds T, Lewis LK, Martin-Fernández JA, Katzmarzyk PT, Barreira T, et al. Health-Related Quality of Life and Lifestyle Behavior Clusters in School-Aged Children from 12 Countries. J Pediatr. abril de 2017;183:178-183.e2.

9. Dumuid D, Olds T, Martín-Fernández JA, Lewis LK, Cassidy L, Maher C. Academic Performance and Lifestyle Behaviors in Australian School Children: A Cluster Analysis. Health Educ Behav. dezembro de 2017;44(6):918–27.

10. Dumuid D, Olds T, Lewis LK, Martin-Fernández JA, Barreira T, Broyles S, et al. The adiposity of children is associated with their lifestyle behaviours: a cluster analysis of school-aged children from 12 nations: Children’s adiposity relates to their lifestyle behaviours. Pediatr Obes. fevereiro de 2018;13(2):111–9.

11. Fernández-Alvira JM, De Bourdeaudhuij I, Singh AS, Vik FN, Manios Y, Kovacs E, et al. Clustering of energy balance-related behaviors and parental education in European children: the ENERGY-project. Int J Behav Nutr Phys Act. 2013;10(1):5.

12. Ferrar K, Golley R. Adolescent Diet and Time Use Clusters and Associations With Overweight and Obesity and Socioeconomic Position. Health Educ Behav. junho de 2015;42(3):361–9.

13. Knebel MTG, Matias TS, Lopes MVV, Dos Santos PC, Da Silva Bandeira A, Da Silva KS. Clustering of Physical Activity, Sleep, Diet, and Screen-Based Device Use Associated with Self-Rated Health in Adolescents. Int J Behav Med. outubro de 2022;29(5):587–96.

14. Magee CA, Caputi P, Iverson DC. Patterns of health behaviours predict obesity in Australian children: Health profiles and obesity. J Paediatr Child Health. abril de 2013;49(4):291–6.

15. Miguel-Berges ML, Zachari K, Santaliestra-Pasias AM, Mouratidou T, Androutsos O, Iotova V, et al. Clustering of energy balance-related behaviours and parental education in European preschool children: the ToyBox study. Br J Nutr. 28 de dezembro de 2017;118(12):1089–96.

16. Moraes ACF, Fernández-Alvira JM, Rendo-Urteaga T, Julián-Almárcegui C, Beghin L, Kafatos A, et al. Effects of clustering of multiple lifestyle-related behaviors on blood pressure in adolescents from two observational studies. Prev Med. janeiro de 2016;82:111–7.

17. Moschonis G, Mavrogianni C, Karatzi K, Iatridi V, Chrousos GP, Lionis C, et al. Increased physical activity combined with more eating occasions is beneficial against dyslipidemias in children. The Healthy Growth Study. Eur J Nutr. abril de 2012;52(3):1135–44.

18. Moschonis G, Kalliora AC, Costarelli V, Papandreou C, Koutoukidis D, Lionis C, et al. Identification of lifestyle patterns associated with obesity and fat mass in children: the Healthy Growth Study. Public Health Nutr. março de 2013;17(3):614–24.

19. Nuutinen T, Lehto E, Ray C, Roos E, Villberg J, Tynjälä J. Clustering of energy balance-related behaviours, sleep, and overweight among Finnish adolescents. Int J Public Health. novembro de 2017;62(8):929–38.

20. Pereira S, Katzmarzyk P, Gomes T, Borges A, Santos D, Souza M, et al. Profiling Physical Activity, Diet, Screen and Sleep Habits in Portuguese Children. Nutrients. 2 de junho de 2015;7(6):4345–62.

21. Pérez-Rodrigo C, Gil Á, González-Gross M, Ortega R, Serra-Majem L, Varela-Moreiras G, et al. Clustering of Dietary Patterns, Lifestyles, and Overweight among Spanish Children and Adolescents in the ANIBES Study. Nutrients. 28 de dezembro de 2015;8(1):11.

22. Saldanha-Gomes C, Marbac M, Sedki M, Cornet M, Plancoulaine S, Charles MA, et al. Clusters of diet, physical activity, television exposure and sleep habits and their association with adiposity in preschool children: the EDEN mother-child cohort. Int J Behav Nutr Phys Act. dezembro de 2020;17(1):20.

23. Wiersma R, Rijnks RH, Bocca G, Boezen HM, Hartman E, Corpeleijn E. Regional variation in lifestyle patterns and BMI in young children: the GECKO Drenthe cohort. Int J Health Geogr. dezembro de 2022;21(1):7.
